# Inherited cobalamin malabsorption. Mutations in three genes reveal functional and ethnic patterns

**DOI:** 10.1186/1750-1172-7-56

**Published:** 2012-08-28

**Authors:** Stephan M Tanner, Amy C Sturm, Elizabeth C Baack, Sandya Liyanarachchi, Albert de la Chapelle

**Affiliations:** 1 Human Cancer Genetics, Comprehensive Cancer Center, The Ohio State University, BRT 850, 460W. 12th Ave, Columbus, OH, 43210, USA; 2 Human Genetics, Department of Internal Medicine, The Ohio State University, Columbus, OH, 43210, USA

**Keywords:** Vitamin B_12_, Cobalamin, Hereditary cobalamin malabsorption, Amnionless, Gastric intrinsic factor, Cubilin, Ancestry, Genetic testing, Founder mutation, Genetic heterogeneity

## Abstract

**Background:**

Inherited malabsorption of cobalamin (Cbl) causes hematological and neurological abnormalities that can be fatal. Three genes have been implicated in Cbl malabsorption; yet, only about 10% of ~400-500 reported cases have been molecularly studied to date. Recessive mutations in *CUBN* or *AMN* cause Imerslund-Gräsbeck Syndrome (IGS), while recessive mutations in *GIF* cause Intrinsic Factor Deficiency (IFD). IGS and IFD differ in that IGS usually presents with proteinuria, which is not observed in IFD. The genetic heterogeneity and numerous differential diagnoses make clinical assessment difficult.

**Methods:**

We present a large genetic screening study of 154 families or patients with suspected hereditary Cbl malabsorption. Patients and their families have been accrued over a period spanning >12 years. Systematic genetic testing of the three genes *CUBN*, *AMN*, and *GIF* was accomplished using a combination of single strand conformation polymorphism and DNA and RNA sequencing. In addition, six genes that were contenders for a role in inherited Cbl malabsorption were studied in a subset of these patients.

**Results:**

Our results revealed population-specific mutations, mutational hotspots, and functionally distinct regions in the three causal genes. We identified mutations in 126/154 unrelated cases (82%). Fifty-three of 126 cases (42%) were mutated in *CUBN*, 45/126 (36%) were mutated in *AMN*, and 28/126 (22%) had mutations in *GIF*. We found 26 undescribed mutations in *CUBN*, 19 in *AMN*, and 7 in *GIF* for a total of 52 novel defects described herein. We excluded six other candidate genes as culprits and concluded that additional genes might be involved.

**Conclusions:**

Cbl malabsorption is found worldwide and genetically complex. However, our results indicate that population-specific founder mutations are quite common. Consequently, targeted genetic testing has become feasible if ethnic ancestry is considered. These results will facilitate clinical and molecular genetic testing of Cbl malabsorption. Early diagnosis improves the lifelong care required by these patients and prevents potential neurological long-term complications. This study provides the first comprehensive overview of the genetics that underlies the inherited Cbl malabsorption phenotype.

## Background

The metabolic pathway of vitamin B_12_ (Cobalamin, Cbl) was elucidated by studying rare disorders in children [1,2]. Deficiency of vitamin B_12_ (Cobalamin, Cbl) in childhood is usually caused by chronic malnutrition, parasitic infections, or genetic defects. With the advent of modern agriculture and medicine, the first two causes have largely disappeared, although they may persist in less developed regions of the world or among individuals who practice unbalanced dietary habits [3]. Genetic defects in Cbl absorption, serum transport, and intracellular metabolism are found worldwide [1,4,5]. Clinical symptoms may be present at birth for intracellular defects (complementation groups cblA OMIM251100; cblB OMIM251110; cblC OMIM277400; cblD OMIM277410; cblE OMIM236270; cblF OMIM277380; cblG OMIM 250940) and transcobalamin 2 deficiency (OMIM275350). However, in the case of intestinal Cbl malabsorption, obvious signs emerge usually only after several months or even years, when the fetal supply stored in the liver has been exhausted [6], and some adolescent cases have been noted [7,8]. The signs of Cbl malabsorption are general weakness, slow growth, developmental delays and learning difficulties, dementia, psychological problems, neurodegeneration of the spinal cord, increased rate of infections due to neutropenia, thrombocytopenia, and megaloblastic anemia with lethal consequences if not treated [9]. Clinical diagnostic markers include low serum Cbl, elevated homocysteine and methylmalonic acid in serum or urine, and exclusion of antibodies against parietal cells and gastric intrinsic factor (IF), whose presence would indicate pernicious anemia. But none of these tests is specific for Cbl malabsorption [10]. The Schilling test [11], measuring the absorption of radio-labeled Cbl, was prematurely retired without an adequate replacement [12]. Ultimately, the final diagnosis is reached by exclusion of many differential diagnoses and can only be confirmed by genetic testing. On the other hand, treatment is often administered via parenteral Cbl supplementation without confirming the diagnosis [9].

The knowledge of the genetic basis of hereditary Cbl malabsorption has much improved over the past decade, with three genes now implicated in its etiology. In 1999, the gene *CUBN* encoding cubilin was found mutated in a series of Finnish patients with selective malabsorption of Cbl and proteinuria [13], followed by the gene *AMN* that encodes amnionless, which was found mutated in several Norwegian and Jewish patients [14,15]. Recessive mutations in either of these two genes cause the disease known as Imerslund-Gräsbeck syndrome (IGS, megaloblastic anemia 1; OMIM261100) or selective vitamin B_12_ malabsorption with proteinuria [16-19]. Cubilin and amnionless form the cubam dimer, which functions as the ileal receptor for the gastric intrinsic factor and Cbl complex (IF-B_12_) that is responsible for uptake of the essential food-born vitamin [20,21]. A clinically similar disease without proteinuria is gastric intrinsic factor deficiency (IFD, OMIM261000 [22]) due to recessive mutations in *GIF*[23-25]. The two-stage Schilling test [11] distinguishes IGS from IFD by the addition of IF in the second assay step, which corrects the malabsorption of Cbl in IFD but not in IGS. However, this test is rarely used today [12] and for clinical purposes the differentiation is usually not possible.

With the implication of these three genes, the majority of IGS and IFD can be reliably diagnosed by genetic testing. However, the diagnostic work is daunting given the genetic heterogeneity and therefore many differential diagnoses have to be excluded before one embarks on genetic testing. In addition, the size of *CUBN*, which consists of 67 exons, and *AMN*, which has proved difficult to analyze, complicate the task. Not surprisingly, of about 400–500 patients reported since 1960, only some 10% were genetically tested and many reports include only a few patients [8,26-36]. This situation has resulted in an incomplete genetic picture of intestinal Cbl malabsorption and hampers not only patient care but future research as well.

We present the results of systematic genetic testing in hereditary intestinal Cbl malabsorption among 154 consecutively recruited sibships or patients. We report 52 previously undescribed mutations in *CUBN*, *AMN*, and *GIF* and discuss the mutational spectrum in various regions of the world, the genetic testing strategy, functional consequences, and suggest that not all responsible genes have been identified yet.

## Subjects and methods

### Patients

We studied patients from all over the world. Both parents were available for study in 90 cases, one parent each in 15 cases, and none in 49 cases (Table 1 and Additional file 1). The diagnosis of hereditary deficiency of vitamin B_12_ absorption was made based on established criteria [6,16,19], usually but not always in tertiary level hospitals. Patients were typically in the range of 6 months to 5 years of age when first diagnosed with Cbl deficiency, however, several patients were over 5 years old before they displayed chronic health problems. Clinical and laboratory details on work-up, exclusion of differential diagnoses, symptom management, and therapy varied widely according to country and treatment center. Low serum Cbl (<200 pg/ml) was the most commonly used marker of Cbl deficiency, sometimes combined with proteinuria, after exclusion of intestinal parasites and nutritional deficiencies. Only very few cases ever had a Schilling test (Additional file 1).

**Table 1:** Genetic study results of 154 patients/families with suspected Cbl malabsorption

**Identifier**^**a**^	**DNA mutation**^**b**^	**Genotype**^**c**^	**predicted consequence mRNA or protein level**^**d**^	**Interpretation**
DT	*CUBN* c.250C>T	hom	p.Gln84*	IGS
MGA47	*CUBN* c.252+1G>A & del ~90 kb proximal of 5'-end to Intron 28	hom	splice site mutation & partial gene deletion	IGS
Fam SA	*CUBN* c.434G>A	hom	p.Gly145Gln	IGS
ZX-1	*CUBN* c.434G>A	hom	p.Gly145Gln	IGS
MGA57	*CUBN* c.489G>A & c.1530G>A	comp het	c.489_490ins137bp; p.Gly164fs & Exon 13 skipping; p.Val473fs	IGS
Norge 1	*CUBN* c.673T>A	hom	p.Cys225Ser	IGS
MGA53	*CUBN* c.889C>T & c.1010C>T	comp het	p.Gln297* & p.Pro337Leu	IGS
MGA1	*CUBN* c.1010C>T & c.2673C>A	comp het	p.Pro337Leu & p.Cys891*	IGS
MGA20	*CUBN* c.1010C>T & del >150 kb proximal of 5'-end to ~150 kb distal of 3'-end	comp het	p.Pro337Leu & complete gene deletion	IGS
MGA29	*CUBN* c.1436C>G & del >150 kb proximal of 5'-end to >160 kb distal of 3'-end	comp het	p.Leu479* & complete deletion	IGS
HS98	*CUBN* c.1526delG & c.1865delC	comp het	p.Gly509fs & p.Thr621fs	IGS
MGA34	*CUBN* c.1838delG & c.3890C>T	comp het	p.Gly613fs & p.Pro1297Leu	IGS
KT	*CUBN* c.1951C>T	hom	p.Arg651*	IGS
Taiwan 1	*CUBN* c.1951C>G & ?	comp het	p.Arg651Gly (rs182512508) & ?	IGS
MGA11	*CUBN* c.2068A>G & c.3330-439C>G	comp het	p.Ile690Val & aberrant splicing	IGS
MGA66	*CUBN* c.2486C>T & ?	comp het	p.Ser829Leu & ?	IGS
MGA76	*CUBN* c.2511_2529del19bp & c.4168G>A	comp het	p.Pro837fs & p.Gly1390Ser	IGS
MGA3	*CUBN* c.2594G>A	hom	p.Ser865Asn	IGS
MGA43	*CUBN* c.2594G>A & ?	comp het	p.Ser865Asn & ?	IGS
MT2	*CUBN* c.2594G>A & c.3749C>T	comp het	p.Ser865Asn & p.Ser1250Phe	IGS
4655-2590	*CUBN* c.2614_2615delGA	hom	p.Asp872fs	IGS
MGA78	*CUBN* c.2614_2615delGA	hom	p.Asp872fs	IGS
MGA56	*CUBN* c.2949C>A	hom	p.Tyr983*	IGS
MGA14	*CUBN* c.3056C>G	hom	p.Ser1019*	IGS
MGA26	*CUBN* c.3096delT & ?	comp het	p.Thr1032* & ?	IGS
MGA7	*CUBN* c.3300-439C>G	hom	aberrant splicing	IGS
RL02	*CUBN* c.3577T>G	hom	p.Trp1193Gly	IGS
FM1(20 cases)	*CUBN* c.3890C>T	hom	p.Pro1297Leu	IGS
AT01	*CUBN* c.3890C>T	hom	p.Pro1297Leu	IGS
MGA17	*CUBN* c.3890C>T	hom	p.Pro1297Leu	IGS
MGA72	*CUBN* c.3890C>T	hom	p.Pro1297Leu	IGS
MGA65	*CUBN* c.3999C>A & ?	comp het	p.Cys1333* & ?	IGS
KA95	*CUBN* c.4115C>G	hom	p.Thr1372Arg	IGS
MGA2	*CUBN* c.4115C>G	hom	p.Thr1372Arg	IGS
Fam A	*AMN* c.14delG	hom	p.Gly5fs	IGS
Fam C	*AMN* c.14delG	hom	p.Gly5fs	IGS
Fam D	*AMN* c.14delG	hom	p.Gly5fs	IGS
Norge 2	*AMN* c.14delG	hom	p.Gly5fs	IGS
MGA12	*AMN* c.43+1G>T & c.701G>T	comp het	splice site mutation & p.Cys234Phe	IGS
MGA88	*AMN* c.43+4A>G & c.100delG	comp het	splice site mutation & p.Ala34fs	IGS
MGA5	*AMN* c.44-3C>G	hom	splice site mutation	IGS
Fam K	*AMN* c.122C>T	hom	p.Thr41Ile	IGS
MGA51	*AMN* c.122C>T & c.1118_1119insCGCT	comp het	p.Thr41Ile & p.Leu374fs	IGS
MGA77	*AMN* c.176T>C	hom	p.Leu59Pro	IGS
FT	*AMN* c.208-1G>C	hom	splice site mutation	IGS
Fam M	*AMN* c.208-2A>G	hom	Exon 4 skipping	IGS
CT	*AMN* c.208-2A>G	hom	Exon 4 skipping	IGS
ET	*AMN* c.208-2A>G	hom	Exon 4 skipping	IGS
MT	*AMN* c.208-2A>G	hom	Exon 4 skipping	IGS
Jor 8.7	*AMN* c.208-2A>G	hom	Exon 4 skipping	IGS
Jor 7.7	*AMN* c.208-2A>G	hom	Exon 4 skipping	IGS
Fam C89	*AMN* c.208-2A>G	hom	Exon 4 skipping	IGS
Israel I	*AMN* c.208-2A>G	hom	Exon 4 skipping	IGS
Israel II	*AMN* c.208-2A>G	hom	Exon 4 skipping	IGS
MGA30	*AMN* c.208-2A>G	hom	Exon 4 skipping	IGS
MGA45	*AMN* c.208-2A>G	hom	Exon 4 skipping	IGS
MGA52	*AMN* c.208-2A>G	hom	Exon 4 skipping	IGS
MGA58	*AMN* c.208-2A>G	hom	Exon 4 skipping	IGS
MGA59	*AMN* c.208-2A>G	hom	Exon 4 skipping	IGS
MGA69	*AMN* c.208-2A>G	hom	Exon 4 skipping	IGS
MGA75	*AMN* c.208-2A>G	hom	Exon 4 skipping	IGS
MGA22	*AMN* c.295delG	hom	p.Gly98fs	IGS
MGA37	*AMN* c.468_469insT & c.1006+34_48del15bp	comp het	p.Gly157fs & Exon 9 skipping	IGS
BT	*AMN* c.514-34G>A	hom	new splice site leading to c.513_514ins32bp; p.Thr172fs	IGS
MGA83	*AMN* c.663G>A	hom	p.Trp221*	IGS
Fam AK	*AMN* c.683_730del48bp	hom	p.Gln228_Leu243del	IGS
PT	*AMN* c.761G>A	hom	p.Gly254Glu	IGS
MGA19	*AMN* c.967_(1169+15)del296bp & c.977_978insCCCG	comp het	partial gene deletion & p.Arg326fs	IGS
MGA86	*AMN* c.1006+16_30del15bp	hom	unknown	IGS
Sudan 1	*AMN* c.1006+34_48del15bp	hom	Exon 9 skipping	IGS
MGA8	*AMN* c.1006+34_48del15bp	hom	Exon 9 skipping	IGS
MGA82	*AMN* c.1006+34_48del15bp	hom	Exon 9 skipping	IGS
MGA13	*AMN* c.1006+34_48del15bp & c.1314_1315delCA	comp het	Exon 9 skipping & p.His438fs	IGS
Belgium 1	*AMN* c.1006+36_50del15bp & c.1253_1254insA	comp het	unknown & p.Leu419fs	IGS
MGA38	*AMN* c.1014_1021delCCTCGGCG	hom	p.Leu339fs	IGS
MGA73	*AMN* c.1014_1021delCCTCGGCG	hom	p.Leu339fs	IGS
MGA81	*AMN* c.1170-6C>T & ?	comp het	splice site mutation? & ?	IGS?
MGA74	*AMN* c.1257+10C>T	hom	splicing defect?	IGS
MGA68	*AMN* c.1314_1315delCA	hom	p.His438fs	IGS
France 1	*GIF* c.79+1G>A	hom	splice site mutation	IFD
MGA4	*GIF* c.79+1G>A	hom	splice site mutation	IFD
MGA25	*GIF* c.79+1G>A	hom	splice site mutation	IFD
MGA49	*GIF* c.79+1G>A & del Intron 8 to distal of 3'-end	comp het	splice site mutation & partial gene deletion	IFD
MGA79	*GIF* c.79+1G>A & c.137C>T	comp het	splice site mutation & p.Ser46Leu	IFD
MGA67	*GIF* c.79+1G>A & c.290T>C	comp het	splice site mutation & p.Met97Thr	IFD
MGA64	*GIF* c.79+1G>A & c.673A>C	comp het	splice site mutation & p.Ser225Arg	IFD
Kuwait 1	*GIF* c.80-1G>A	hom	splice site mutation	IFD
Kuwait 2	*GIF* c.80-1G>A	hom	splice site mutation	IFD
IT	*GIF* c.137C>T	hom	p.Ser46Leu	IFD
NT	*GIF* c.137C>T	hom	p.Ser46Leu	IFD
LT	*GIF* c.161delA	hom	p.Asn54fs	IFD
Fam 8	*GIF* c.183_186delGAAT	hom	p.Met61fs	IFD
MGA33	*GIF* c.183_186delGAAT	hom	p.Met61fs	IFD
MGA55	*GIF* c.183_186delGAAT	hom	p.Met61fs	IFD
MGA39	*GIF* c.183_186delGAAT & c.659T>C	comp het	p.Met61fs & p.Ile220Thr	IFD
MGA27	*GIF* c.256+2T>G & c.659T>C	comp het	splice site mutation & p.Ile220Thr	IFD
MGA35	*GIF* c.290T>C & ?	comp het	p.Met97Thr & ?	IFD
MGA54	*GIF* c.431_438delAGAAGAAC & c.974_975insG	comp het	p.Gln144fs & p.Val325fs	IFD
MGA48	*GIF* c.469T>C & ?	comp het?	p.Phe157Leu & ?	IFD?
HT	*GIF* c.685G>A	hom	p.Ala229Thr	IFD
MGA36	*GIF* c.685G>A	hom	p.Ala229Thr	IFD
D2914	*GIF* c.938C>T & ?	comp het	p.Thr313Ile & ?	IFD
MGA24	*GIF* c.1073+5G>A	hom	splice site mutation	IFD
MGA63	*GIF* c.1073+5G>A	hom	splice site mutation	IFD
MGA92	*GIF* c.1073+5G>A	hom	splice site mutation	IFD
AT	*GIF* c.1175_1176insT	hom	p.Thr393fs	IFD
MGA61	*GIF* c.1222G>A	hom	p.Glu408Lys	IFD
MGA9	*LMBRD1* c.404delC & c.1056delG	comp het	p.Thr135fs & p.Leu352fs	cblF defect
JCA1	*AMN/CUBN* excluded; *GIF/FUT2/CD320/ABCC1/LMBRD1* screened	n/a	n/a	differential diagnosis?
MGA6	*AMN/CUBN/GIF/FUT2/CD320/ABCC1/LMBRD1/TCN2* screened	n/a	n/a	differential diagnosis?
MGA10	*AMN/CUBN/GIF/FUT2/CD320/ABCC1/LMBRD1* screened	n/a	n/a	differential diagnosis?
MGA15	*AMN/CUBN/GIF/FUT2/CD320/ABCC1/LMBRD1* screened	n/a	n/a	differential diagnosis?
MGA16	*AMN/CUBN/GIF/FUT2/CD320/ABCC1/LMBRD1* screened	n/a	n/a	differential diagnosis?
MGA18	*AMN/CUBN/GIF/FUT2/CD320/ABCC1/LMBRD1* screened	n/a	n/a	differential diagnosis?
MGA21	*AMN/CUBN/GIF/FUT2/CD320/ABCC1/LMBRD1* screened	n/a	n/a	differential diagnosis?
MGA23	*AMN/CUBN/GIF/FUT2/CD320/ABCC1/LMBRD1/TCN2* screened	n/a	n/a	differential diagnosis?
MGA28	*AMN/CUBN/GIF/FUT2/CD320/ABCC1/LMBRD1* screened	n/a	n/a	differential diagnosis?
MGA31	*AMN/CUBN/GIF/FUT2/CD320/ABCC1/LMBRD1* screened	n/a	n/a	differential diagnosis?
MGA32	*AMN/CUBN/GIF/FUT2/CD320/ABCC1/LMBRD1* screened	n/a	n/a	differential diagnosis?
MGA40	*AMN/CUBN/GIF* excluded; *FUT2/CD320/ABCC1/LMBRD1* screened	n/a	n/a	differential diagnosis?
MGA41	*AMN/CUBN/GIF* excluded; *FUT2/CD320/ABCC1/LMBRD1* screened	n/a	n/a	differential diagnosis?
MGA42	*AMN/CUBN/GIF* screened	n/a	n/a	differential diagnosis?
MGA44	*AMN/CUBN/GIF/ABCC1/LMBRD1* screened	n/a	n/a	differential diagnosis?
MGA46	*CUBN/GIF* excluded; *AMN/FUT2/CD320/ABCC1/LMBRD1* screened	n/a	n/a	differential diagnosis?
MGA50	*AMN/CUBN/GIF* screened	n/a	n/a	differential diagnosis?
MGA62	*AMN/CUBN/GIF/FUT2/CD320/ABCC1/LMBRD1/TCN1/TCN2* screened	n/a	n/a	*TCN1* defect?
MGA70	*AMN/CUBN/GIF/ABCC1/LMBRD1* screened	n/a	n/a	differential diagnosis?
MGA71	*AMN/CUBN/GIF* screened	n/a	n/a	differential diagnosis?
MGA80	*AMN/CUBN/GIF/ABCC1/LMBRD1* screened	n/a	n/a	differential diagnosis?
MGA84	*AMN/CUBN/GIF* screened	n/a	n/a	differential diagnosis?
MGA85	*AMN* excluded; *CUBN/GIF* screened	n/a	n/a	differential diagnosis?
MGA87	*AMN/CUBN/GIF* screened	n/a	n/a	differential diagnosis?
MGA89	*GIF* excluded; *AMN/CUBN* screened	n/a	n/a	differential diagnosis?
MGA90	*AMN/CUBN* excluded; *GIF* screened	n/a	n/a	differential diagnosis?
MGA91	*TCN1* c.747+3A>C & ?; *GIF* screened	comp het?	splice site mutation?	*TCN1* defect?

^a^Cases are ordered by the location of the mutations in the three genes *CUBN*, *AMN*, and *GIF*, followed by potential differential diagnoses by case code. Additional file 1 online contains a sortable Excel table with additional information.

^b^Numbering relative to adenine in the first ATG start codon of *CUBN* (GenBank RefSeq: NM001081.2), *AMN* (GenBank RefSeq: NM030943.1), and *GIF* (GenBank accession NM005142.2).

^c^hom means homozygous, comp het means compound heterozygous, n/a means not applicable.

^d^Numbering relative to the first methionine deduced from the cDNA sequences. Mutations which seemingly caused a frameshift were described as to where the frameshift occurred rather than when the next stop codon was predicted. Where available experimentally confirmed splicing defects on the mRNA level are listed (for details see text).

### Patient samples

Blood samples for DNA or RNA isolation were obtained after informed consent with prior Institutional Review Board approval (OSU protocol 2005 H0201) according to the Declaration of Helsinki. DNA isolation was performed by standard proteinase K digest, phenol-chloroform extraction and EtOH precipitation at the Ohio State University or locally using commercially available DNA isolation kits from various companies. Total RNA was isolated using the Trizol protocol according to the manufacturer’s instructions (Invitrogen, Carlsbad, CA).

### Mutation screening and genetic analyses

We amplified individual exons of *CUBN* (GenBank RefSeq: NM001081.2), *AMN* (GenBank RefSeq: NM030943.1), and *GIF* (GenBank accession NM005142.2) from genomic DNA by PCR and analyzed the PCR amplicons by single strand conformation polymorphism (SSCP, [37]) and direct DNA sequencing. Sequencing was performed on an ABI PRISM® 3730 DNA analyzer (Applied Biosystems, Foster City, CA). PCR and SSCP conditions and primer sequences are available under a collaborative agreement. All nucleotide numbering is relative to the adenine in the first ATG start codon of the three genes, while the amino acid residue numbering is relative to the first methionine deduced from these cDNA sequences according to standard mutation nomenclature [38]. All exons were sequenced in at least 100 anonymized controls from various ethnic backgrounds (89% Caucasian, 10% African-American, 1% other; [39]. Missense changes were studied for conservation using HomoloGene (Additional file 2) and the PolyPhen-2 program [40]. The genes *TCN1TCN2FUT2CD320LMBRD1*, and *ABCC1* were screened by DNA sequencing in a selected group of patients (Additional file 1).

### Transcript analysis

Suspected splice site changes were studied by comparing them to consensus sequences in spliceDB [41]. If RNA was available, first-strand cDNA was produced from 0.5-1 μg total RNA using the AMV cDNA Synthesis Kit (Roche Applied Science, Indianapolis, IN) according to the instructions with a poly dT_24_-primer. Subsequent reverse-transcription-PCR was performed with assorted cDNA primers covering the desired regions within *CUBN* (GenBank RefSeq: NM001081.2), *AMN* (GenBank RefSeq: NM030943.1), or *GIF* (GenBank accession NM005142.2). DNA sequencing was performed as above.

## Results

### Nature and frequency of the mutations

**Figure 1:**
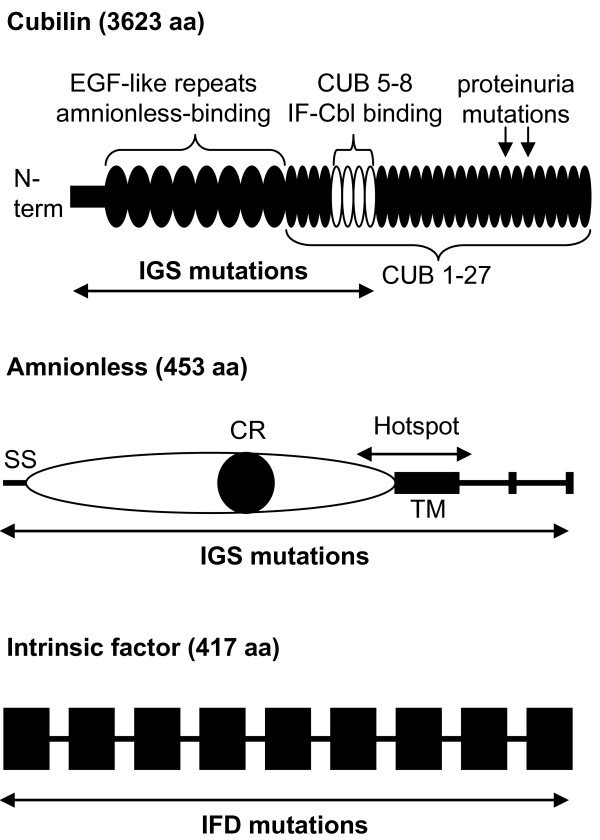
**Illustration of the proteins cubilin and amnionless mutated in IGS and intrinsic factor mutated in IFD, leading to inherited cobalamin malabsorption.** Mutations that cause IGS in cubilin were restricted to exons 1–28 that encode the amnionless binding domain (EGF1-8) and the IF-Cbl binding region (CUB5-8). Two other mutations located towards the carboxy-terminal end (p.Ser2785fs in CUB20 and p.Ile2984Val in CUB22) caused proteinuria. Mutations in amnionless and intrinsic factor were located throughout the protein and many mutations affect splicing (Table 1). The mutational hotspot in *AMN* includes the transmembrane domain and flanking GC-rich repetitive genomic sequences that are apparently unstable (see text). CUB means complement C1r/C1s, Uegf, and Bone morphogenic protein-1, EGF means epidermal growth factor repeats, IF means intrinsic factor, CR means cysteine-rich domain, SS means signal sequence (aa 1–19), TM means transmembrane domain (aa 360–380), and aa means amino acid. The proteins are not drawn to scale.

We have identified mutations in 126 of 154 (82%) cases or families that were ostensibly unrelated (Table 1 and Figure 1; Additional files 1 &2). Of these 126 cases, 53 (42%) were mutated in *CUBN*, 45 (36%) were mutated in *AMN*, and 28 (22%) had mutations in *GIF*. We analyzed both parents in 75 cases and one parent in eight cases and positive carrier status in all parents was established, excluding any *de novo* mutations. Parental samples were unavailable for 43 cases. In nine cases only one mutation was identified (see missing mutations). One case (MGA9) was initially classified as IGS, based on a false-positive Schilling test, but later turned out to be mutated in the gene *LMBRD1* coding for a lysosomal Cbl exporter (*cblF* defect; patient 9 in [42]). Twenty-seven cases (18%) remain unresolved and sequencing of *TCN1TCN2FUT2CD320LMBRD1*, and *ABCC1* in selected cases identified no additional mutations (Additional file 1).

Several intronic and suspected silent changes caused splicing defects (*AMN* c.514-34G>A and c.1006+34_48del15bp; *CUBN* c.489G>A and c.1530G>A) and thus predictions of mRNA processing based on sequence changes might deviate from reality (Table 1). Consequently, mutations which seemingly caused a frameshift were described as to where the frameshift occurred rather than when the next stop codon was predicted, unless the consequences on the mRNA level were studied.

### Mutations in *CUBN*

We have identified 30 *CUBN* point mutations and three large deletions (Table 1 and Additional file 1) in 53 cases or families. Of these 33 different gene defects, seven were previously reported, while 26 novel changes are presented here.

The most common *CUBN* mutation was missense change c.3890C>T; p.Pro1297Leu, a Finnish founder mutation in exon 27 [13,43]. It occurred mostly in homozygous state but its relatively high incidence among the Finns (>25 families or cases) also unraveled two other rare *CUBN* mutations c.1838delG; p.Gly613fs in MGA34 and c.1230+1G>A [15].

Our results showed that several other ethnicity-specific mutations exist in *CUBN*. Two Saudi Bedouin families shared missense change c.434G>A; p.Gly145Glu (Fam SA in [15] and ZX-1). Missense mutation c.1010C>T; p.Pro337Leu was found three times in combination with other defects in cases of German (MGA53 with p.Gln297* and MGA20 with a large deletion) and Western European origin (case MGA1 with p.Cys891*), suggesting that p.Pro337Leu is a Germanic mutation. Intronic mutation c.3330-439C>G is Swedish in origin (homozygous in patient MGA7 and case FM2 in [13]; compound in MGA 11 with p.Ile690Val). Furthermore, Ashkenazi frameshift mutation c.2614_2615delGA (cases 4655–2590 and MGA78) and Turkish missense mutation c.4115C>G; p.Thr1372Arg (families KA95 and MGA2) were found twice each homozygously. However, patients from multi-ethnic Turkey carried several different IGS and IFD mutations (Table 1 and Additional file 1).

Missense change c.2594G>A; p.Ser865Asn occurred in Albania (family MGA3, homozygous), Turkey (case MT2 with p.Ser1250Phe), and in a Scottish case in whom the second mutation is still undetected (MGA43). This is the only IGS or IFD mutation we have encountered in one anonymized control individual. However, p.Ser865Asn was heterozygous in that individual, who had no other suspicious changes. Missense change p.Ser865Asn was found at low frequency in the NIH Exome Sequencing Project (rs138083522, A-allele frequency 0.014). Ser865 is not 100% conserved among mammals and p.Ser865Asn was considered a benign amino acid change (PolyPhen-2 score = 0.007). Thus, its functional relevance is unknown. However, it was seen in four patients, homozygously in two siblings of family MGA3 and once in combination with the damaging mutation p.Ser1250Phe, which suggests that missense p.Ser865Asn is a pathogenic IGS mutation or at least in linkage disequilibrium with an undetected *CUBN* mutation.

Of the remaining 20 *CUBN* mutations, 15 are clearly deleterious: two large deletions, seven nonsense, one splice site, and three frameshift mutations. The two alleged silent mutations in individual MGA57 each affected the last nucleotide in exons 5 and 13, respectively. Reverse-transcription-PCR revealed that c.489G>A (exon 5) caused retention of part of intron 5 (137 bp) and c.1530G>A (exon 13) led to the skipping of exon 13 (113 bp), causing a frameshift in both alleles (p.Gly164fs and p.Val473fs).

Of the 5 remaining missense changes, p.Gly1390Ser occurred in combination with a frameshift mutation in family MGA76. Similarly, p.Cys225Ser (patient Norge 1) and p.Trp1193Gly (patient RL02) targeted residues that are 100% conserved from humans to *C. elegans* and were considered damaging by PolyPhen-2, supporting their pathogenic role. The other two missense mutations p.Arg651Gly (family Taiwan 1) and p.Ser829Leu (MGA66) are discussed further below.

### Mutations in *AMN*

In total we have detected 27 different *AMN* mutations of which 19 were previously undescribed (Table 1 and Additional file 1). The most frequent mutation is c.208-2A>G, which causes an out-of-frame loss of exon 4 in the mRNA [14]. This ancient founder mutation is about 13,600 years old [44] and causes some 15% of IGS cases worldwide and more than 50% among Turks, Jordanians, and Sephardim combined, many of them expatriates. It accounted for 16 of the 45 *AMN*-mutated sibships in our cohort. A second acceptor splice site mutation in intron 3 (c.208-1G>C) affected the neighboring nucleotide in family FT.

The second most common mutation is a 15-bp deletion in intron 9 (c.1006+34_48del15bp) that was found in 5 sibships, in two of them combined with other mutations (MGA37: c.468_469insT, and MGA13: c.1314_1315delCA). At first, we assumed that the 15-bp deletion was a polymorphism. However, non-Mendelian inheritance patterns of flanking markers indicated that in the presence of this mutation, the wildtype allele in heterozygotes failed to amplify. After designing deletion-specific PCR primers, we were able to show that for example in MGA8 both parents were heterozygous and the patient was homozygous for c.1006+34_48del15bp. Follow-up reverse-transcription-PCR analysis and DNA sequencing showed that this particular deletion caused the complete loss of exon 9 (163 bp), leading to a frameshift in the resulting mRNA. The mutation occurred in Southwestern Europe (France and Spain) but was also found in Sudan (family Sudan 1) and in the USA (patient MGA82). The differing flanking haplotypes in the European, American, and Sudanese cases and the fact that two similar 15-bp deletions occurred in patients MGA86 (homozygous c.1006+16_30del15bp) from Yemen and Belgium 1 (compound heterozygous c.1006+36_50del15bp and c.1253_1254insA; [15]) from Europe pointed to a mutational hotspot. Four additional insertion-deletion mutations in the same region accounted for four more cases from Europe (MGA51: c.1118_1119insCGCT with missense c.122C>T; Thr41Ile and MGA19: c.967_(1169+15)del296bp and c.977_978insCCCG) and Central America (MGA38 and MGA73: homozygous c.1014_1021delCCTCGGCG). Moreover, the heterozygous change c.1170-6C>T in intron 10 (patient MGA81) and a homozygous change (c.1257+10C>T, patient MGA74) in intron 11 might affect mRNA processing as seen with other intronic changes in this region but RNA was not available to study them further. The repetitive and GC-rich region extending from intron 8 to intron 11 (838 bp with 75% G+C-content) includes the transmembrane domain in exon 10 (aa ~360-380; [45]).

The remaining 12 *AMN* mutations were private events in individual families or cases, with the exception of c.1314_1315delCA, which was seen in MGA13 and homozygously in MGA68. Case MGA12 carried a donor splice site and a missense mutation as detailed previously [33]. Patient MGA88 was compound heterozygous for a splice site and a frameshift mutation, while the remaining seven sibships were homozygous for the respective mutation: MGA5, MGA77, MGA22, MGA83, Fam AK [14], PT, and BT with intronic point mutation c.514-34G>A. This ostensibly harmless change activated a cryptic splice site that caused the misincorporation of 32 bp in the mRNA (c.513_514ins32bp; p.Thr172fs).

### Mutations in *GIF*

In our cohort 28/126 (22%) carried mutations in *GIF*. A total of 18 different mutations were identified of which 11 were previously reported and 7 are documented here (Table 1 and Additional file 1). The most numerous was splice site mutation c.79+1G>A in intron 1 that was found in seven sibships. Three times it was found homozygously (France 1, MGA4, and MGA25) and four times in combination with other defects: with a 3’-terminal deletion in MGA49 (see below) and with three different missense mutations in MGA79, MGA67, and MGA64, respectively. In patient MGA79 from Siberia it was missense mutation c.137C>T; p.Ser46Leu that was also found in two families from Turkey (IT and NT). Mutation p.Ser46Leu might be a Central Asian founder event but we lack sufficient information to prove that. In patient MGA67, we detected missense change c.290T>C; p.Met97Thr, which was described previously [8] and also occurred in a case from Finland (MGA35). In MGA64, the splice site mutation was compound heterozygous with missense mutation c.673A>C; p.Ser225Arg. Splice site mutation c.79+1G>A is apparently a Western Caucasian founder mutation, as we have not observed variation on the flanking haplotype.

Two more founder mutations were detected in *GIF*: c.183_186delGAAT; p.Met61fs, which is African in origin [23] and Chaldean splice site mutation c.1073+5G>A [46]. While the latter was only found in homozygosity, p.Met61fs once occurred in a mixed African-Caucasian patient (MGA39) together with missense mutation c.659T>C; p.Ile220Thr [23]. Residue Ile220 is conserved among mammals and the mutation scored damaging (PolyPhen-2 score 0.998). This missense change was also found in a second case (MGA27) together with a splice site mutation c.256+2 T>G [30].

Kuwaiti acceptor splice site mutation c.80-1G>A and two private insertion-deletion mutations c.161delA (case LT) and c.1175_1176insT (case AT) were described previously [24] and missense change c.1222G>A; p.Glu408Lys in case MGA61 [32] affected a conserved residue. All these mutations were homozygous.

Families HT from Turkey and MGA36 from Lebanon shared the missense mutation c.685G>A; p.Ala229Thr. MGA54 carried two private frameshift mutations: c.431_438delAGAAGAAC and c.974_975insG and may have had a false positive Schilling test. Families MGA48 and D2914 are described below.

### Large deletions in *CUBN* and *GIF*

Large gene deletions were discovered via incompatible Mendelian inheritance patterns of sequence polymorphisms or microsatellite markers. All four identified deletions were compound heterozygous with point mutations found on the other allele (Table 1 and Additional file 1). Three large deletions were detected in the *CUBN* gene, two removed the complete gene (MGA20 and MGA29), while one deletion removed the 5’-half of the gene up to exon 28 (MGA47). In *GIF*, a partial gene deletion extended from intron 8 past the 3’-end in the two siblings of MGA49. Because of the large physical distances involved we were unable to identify the exact deletion breakpoints via PCR but we have used markers flanking the genes in order to demarcate the deletions.

### Missing mutations

In nine cases, we have found only one likely mutation (one in *AMN*; five in *CUBN*, and three in *GIF*; Table 1 and Additional file 1). Four cases had recurrent mutations seen in other IGS or IFD cases or they were clearly deleterious (MGA43, MGA26, MGA65, and MGA35). The remaining five cases (Taiwan 1, MGA66, MGA81, MGA48, and D2914) carried heterozygous changes that were not encountered among controls or dbSNP with the exception of *CUBN* c.1951C>G; p.Arg651Gly (SNP rs182512508, without frequency information), which was detected in two siblings of family Taiwan 1. The two affected brothers shared the same *CUBN* genotype, while residue Arg651 is 100% conserved among vertebrates and considered detrimental by PolyPhen-2 (score = 1.0). Thus, p.Arg651Gly is likely a pathogenic change. In patient MGA66, the observed missense change *CUBN* c.2486C>T; p.Ser829Leu likewise affected a highly conserved residue which was considered damaging by PolyPhen-2 (score = 1.0). Thus, we concluded that p.Ser829Leu is an IGS mutation.

The change in intron 10 of *AMN* (c.1170-6C>T) in patient MGA81 was found in the aforementioned unstable GC-rich region of *AMN*. It is therefore possible that this change disturbed the mRNA processing as seen with several other intronic changes in this region but RNA was not available to study it further.

In patient MGA48, only *GIF* c.469T>C; p.Phe157Leu was detected as a candidate mutation. The IF residue Phe157Leu is conserved among vertebrates with the exception of the dog, which has a leucine residue in place of phenylalanine, thus its exact functional consequences remain to be studied (PolyPhen-2 score = 0.003). However, it was never detected in any other individual or control other than the older sister of the patient, who is healthy and has a different *GIF* genotype. Thus, p.Phe157Leu is a likely culprit in this Lebanese girl with Cbl deficiency since the other two genes were excluded. Finally, in patient D2914, missense change *GIF* c.938C>T; p.Thr313Ile is likely pathogenic (100% conserved among vertebrates and PolyPhen-2 score = 0.999).

## Discussion

### Genetic defects in intestinal malabsorption

The spectrum of mutations in the three genes *CUBN*, *AMN*, and *GIF* includes nonsense, missense, insertion-deletion, splice site, and intronic mutations, as well as large deletions first reported herein (Table 1 and Additional file 1).

**Figure 2:**
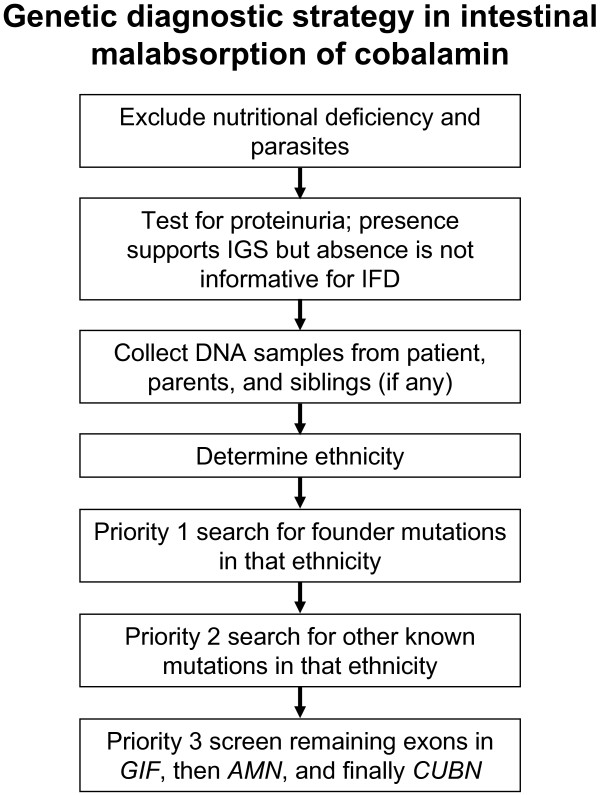
Flow-diagram of the genetic diagnostic strategy in inherited cobalamin malabsorption.

Given that many mutations appear to be private or restricted to a specific ethnicity or population, estimates of the population frequency of IGS or IFD and their underlying mutations are difficult to make and cannot be extrapolated across populations. Thus, we purposely avoided estimating the frequency of IGS or IFD as we believe it will not be sufficiently accurate to be useful. However, many mutations affect specific ethnic groups; as a result, ancestry was demonstrated to facilitate genetic testing [23,44,46]. The genetic heterogeneity led us to develop an ethnicity-focused screening strategy that targets founder mutations first (Figure 2).

#### CUBN

Our analyses of 53 families or cases with 33 *CUBN* mutations show that no IGS mutation was found beyond exon 28 and one of the deletions (case MGA47) covered the same region. This observation is of clinical utility because it suggests that defects beyond exon 28 have no impact on the absorption of Cbl, as long as the protein is stable. In fact, it was shown that a homozygous frameshift mutation in exon 53 of *CUBN* (c.8355delA; p.Ser2785fs) only caused proteinuria [47]. Moreover, a missense variant in exon 57 (c.8950A>G; p.Ile2984Val) was associated with albuminuria [48]. Thus, mutations in *CUBN* cause IGS apparently only when they affect the cubilin-amnionless interaction domain (amino-terminal third of cubilin, exons ~1-20; [21]) or the IF-Cbl binding site (CUB domains 5–8, exons 21–29; [43]; Figure 1). It is possible that genomic deletions in *CUBN* are more common and could account for some of the missing mutations and unresolved cases. In individual patients however, deletions can be difficult to detect unless the deletion is homozygous. It is therefore prudent to include parents and siblings in the genetic analysis since the genetic information (heritable SNPs and other variants) might expose genomic deletions. Technically, multiplex ligation-dependent probe amplification (MLPA) or next-generation sequencing should detect deletions but an MLPA kit for *CUBN* is currently not available and whole-genome sequencing for routine diagnostics is still in its infancy.

#### AMN

Mutations in *AMN* are dominated by founder events and a mutational hotspot in the region of introns 8–11 that includes the transmembrane domain in exon 10 [45]. Its analysis was technically challenging because of a highly repetitive GC-content and required high-quality genomic DNA. Moreover, in individuals heterozygous for *AMN* c.1006+34_48del15bp the wildtype allele dropped-out during PCR; thus in cases that show seemingly non-Mendelian inheritance of rare changes, a detailed molecular follow-up by RT-PCR or various PCR primer combinations is advisable.

#### GIF

A single IFD patient with a homozygous 4-bp deletion in *GIF* was first described in 2004 [25]. The finding of additional mutations in *GIF*[24] was the result of a genome-wide search among patients with suspected IGS that were previously excluded for defects in *CUBN* and *AMN*[15].

While IF is conserved among Amniota (mammals, birds, and reptiles) the level of conservation among lower vertebrates is less clear. This analysis is complicated by the fact that the genes for transcobalamin 2 (*TCN2*) and haptocorrin (transcobalamin 1; *TCN1*) are similar as a result of ancient gene duplication events [1]. The three genes share the same genomic structure with 9 exons and a Cbl binding domain, pointing to a common ancestral gene [49,50]. Despite the coding and structural similarities, PCR-based analysis of three genes, *GIFTCN1*, and *TCN2* has not caused technical problems. Conversely, transcript analysis of *GIF* using RNA derived from blood cells has proved difficult because the gene is not expressed in that tissue. Even successive rounds of PCR with nested primers and have not succeeded in amplifying the *GIF* mRNA. This is not particularly surprising since haptocorrin and transcobalamin are the specific Cbl-transporters in the blood [10], and haptocorrin is also found in saliva [51]. Thus, transcript studies of *GIF* will likely require gastric sampling to obtain the parietal cells that produce IF.

### Genotype-phenotype observations

Because of the limited clinical details that were available from some patients and the fact that many mutations were private, meaningful phenotype-genotype correlations in IGS and IFD were limited. The course of therapy and the health care environment varied widely, as did the age of onset of the symptoms. The most obvious clinical sign, megaloblastic anemia, was not always present and is not unique to IGS or IFD [6]. On the other hand, early hematological and neurological signs can go unnoticed for many months or years. Generally, clinical diagnosis was based on excluding various differential diagnoses, so many patients were only referred for genetic testing several months after acute problems began. However, treatment with parenteral Cbl was often initiated before a firm diagnosis could be made. Thus, the necessary information to predict the age of onset, the degree of manifestations, and the course of the disease depending on the type of mutation is lacking.

Proteinuria is found in many IGS cases [19] but has rarely been seen in IFD except in two cases from our series, MGA67 and MGA79. We suspect that these two cases had proteinuria unrelated to their IFD defects. The root cause of the proteinuria in IGS is due to the fact that mutations in *CUBN* or *AMN* not only prevent the intestinal uptake of Cbl but may also impair the renal reabsorption of proteins [21,52]. Since amnionless is required to localize cubilin to the luminal membrane in the intestines and kidneys [34,53], deleterious mutations in *AMN* often cause simultaneous Cbl deficiency and proteinuria since the cubam complex is no longer able to mediate uptake of its many ligands [52]. In *CUBN*, mutations in the cubilin-amnionless interaction domain (amino-terminal third of cubilin, exons ~1-20; [21]) or total loss of the protein may similarly lead to concurrent Cbl deficiency and proteinuria, while mutations in the IF-Cbl binding site (CUB domains 5–8, exons 21–29; [43]) can cause Cbl deficiency without proteinuria [19]. The finding of mono-symptomatic proteinuria due to mutations in *CUBN* (c.8355delA; p.Ser2785fs; [47] and c.8950A>G; p.Ile2984Val; [48]) pointed to the fact that not all mutations in this gene have the same physiological consequences. Thus, defects in cubilin have pleiotropic effects, e.g. for kidney function [54].

Lack of Cbl not only causes anemia but also impairs neurological function [3]. In young infants, hypotonia, seizures, developmental delay, and brain atrophy often occur during the first six months [55]. In severe cases, the patients can perish during early childhood. In older children, movement disorders, dementia, delirium, or psychosis were observed [56]. One IGS case (MGA12) with mutations in *AMN* showed severe psychosis, which only responded to high-dose Cbl therapy [33]. It was suggested that an active Cbl transport mechanism at the blood–brain barrier exists, and that amnionless may be part of this mechanism. Consequently, it is possible that certain mutations in *CUBN* or *AMN* affect the neurological presentation differently.

Mouse models deficient in *CUBN*[57] or *AMN*[45] have been developed and proved embryonic lethal. Given the deleterious nature of many *CUBN* and *AMN* mutations in humans, it has become clear that the role of cubilin and amnionless in rodent development is distinct from the role that these proteins play in humans. Thus far, it is not obvious what functions cubilin and amnionless have in primate embryogenesis [58]. IGS was also observed in dogs with two different mutations in the canine *AMN* gene and the phenotype was similar to that observed in humans [53]. In an attempt to define the differences between rodents and higher mammals regarding *AMN*[14], we created *Amn* knock-in mice with three different human IGS mutations (data not shown). The high degree of sequence conservation between human and mouse permitted the identical recreation of the human IGS mutations in the mouse. Two of these mutations (*Amn* c.14delG; p.Gly5fs and *Amn* c.683_730del48; p.Gln228_Leu243del) were homozygously lethal, since we never observed any homozygous pups among over 100 offspring in each case (data not shown). On the other hand, the Norwegian missense mutation *Amn* c.122C>T; p.Thr41Ile was viable in the homozygous mouse and without any apparent phenotype (data not shown). Recently, conditional *Cubn* knock-out mice were created [59], which should permit a better definition of the essential role of cubilin in mouse embryogenesis and renal function.

### Other candidate genes

We have screened 27 cases or sibships (18%) for mutations in *CUBNAMN*, and *GIF* without detecting any pathogenic mutations (Table 1). For seven families the involvement of some or all of the genes was genetically excluded based on different genotypes in two affected siblings (JCA1, MGA40, MGA41, MGA46, MGA85, MGA89, and MGA90). Among the 20 remaining single patients, patients MGA62 and MGA91 were thought to suffer from a defect in the *TCN1* gene that encodes haptocorrin. Patient MGA91 carried a novel heterozygous change in intron 5 (*TCN1* c.747+3A>C) that was suspect but no RNA was available to study potential splicing aberrations. So far, two truncating mutations have been described in *TCN1*[60] but to what degree haptocorrin deficiency plays a role in Cbl deficiency remains to be studied.

It is conceivable that we have missed some mutations. These could be located in introns or regulatory sequences distant from the exons. However, many cases carried two distinct alleles of the IGS/IFD genes, thus we would have expected two different disease mutations, which would be less likely to be missed. Overall, we have achieved a sensitivity of 82% in our mutation screening strategy (Figure 2).

Based on their role in transport of Cbl, alternative candidate genes *FUT2*[61-63], *CD320*[64,65], *LMBRD1*[42], and *ABCC1*[66] were screened by DNA sequencing in a selected group of unresolved cases but no mutations were found (Additional file 1 and [67]).

Deficiency of transcobalamin 2 (TC2; OMIM275350) with mutations in *TCN2*[68,69] represents an alternative diagnosis. Symptoms include megaloblastic anemia, diarrhea, vomiting, failure to thrive, recurring infections, and mental retardation. Thus, many clinical features overlap with IGS and IFD, although mental retardation is not usually associated with IGS and IFD. We sequenced a few atypical cases for mutations in *TCN2* (MGA6, MGA23, and MGA62) but found no mutation. In general, TC2 deficiency manifests rapidly in the first 1–2 months after birth and was excluded in most cases before referral for IGS/IFD mutation screening.

**Figure 3:**
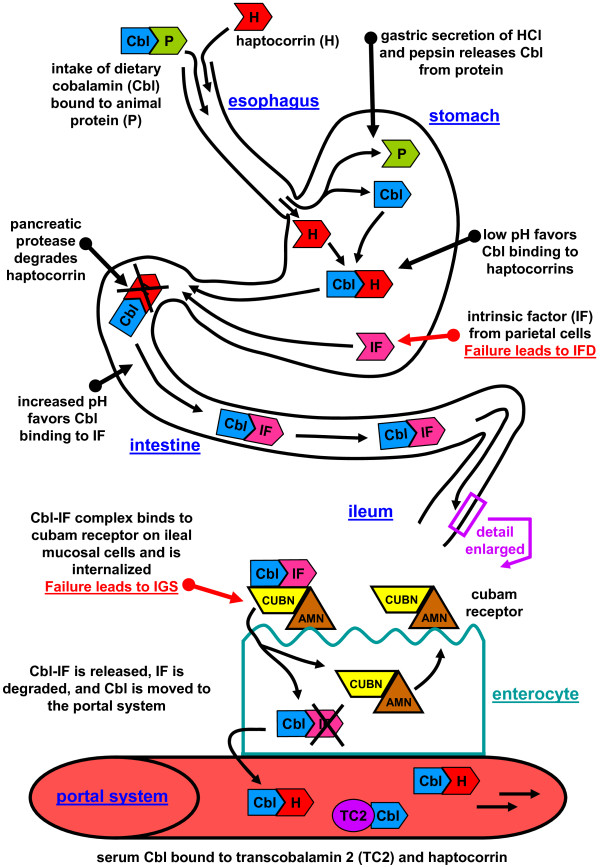
**Release of cobalamin from the food and intestinal uptake.** Cobalamin (Cbl) uptake and transport pathway from food intake to the portal system. Cbl is released from food proteins (P) by salivary and gastric enzymes and then binds to haptocorrin (H). In the proximal intestine, pancreatic enzymes degrade haptocorrin and Cbl binds to intrinsic factor (IF). In the ileum, the Cbl-IF complex binds to the cubam receptor (CUBN-AMN) and enters the enterocyte (ileal epithelia). Inside the cell, IF is degraded and Cbl is moved to the portal system by an unknown process. The cubam receptor is recycled back to the membrane. In the blood, transcobalamin 2 (TC2) transports Cbl to the tissues. The role of haptocorrin carrying 80% of the Cbl serum fraction is unknown.

Accordingly, Cbl pathway genes yet to be identified might explain some of these remaining cases of inherited Cbl deficiency. Based on the documented Cbl uptake pathway (Figure 3), a likely location for failure could be the loading of TC2 with Cbl in the enterocyte to form holo-TC2 or the export of holo-TC2 from of the enterocyte. This particular part of the Cbl transport is not well understood but similarities to the intracellular *cblF* defect (OMIM277380) that affects the lysosomal membrane transport [70] could be postulated.

### Prospective screening recommendations and future prospects

With the gathered information on ethnicity-specific mutations, it has become feasible to perform targeted screening for common or local founder mutations (Figure 2 and Additional file 1). However, human migrations change the genetic make-up of populations and it is important to trace ethnic ancestry cautiously.

The observation that mutations in *CUBN* are limited to the first 28 exons has simplified the genetic analysis (Figure 1) but the need to screen three genes remains unchallenged for now because clinical tests cannot reliably distinguish IGS and IFD. Parallel, whole-exome or whole-genome sequencing using next generation technology might permit concurrent screening of the three genes. However, clinical laboratory regulations and ethical concerns about the additional sequence data produced will delay the routine introduction of the technology. Consequently, Sanger-based exon-by-exon sequencing will remain the method of choice for the coming years to confirm IGS and IFD.

For clinical diagnostics, a new non-radioactive Cbl absorption test may eventually replace the Schilling test [71]. The patient is given a dose of cyano-Cbl, which enters the blood unchanged via the intestine in healthy people but not in cases with IGS or IFD. Then the fraction of transcobalamin-bound cyano-Cbl is measured, which reflects the degree of Cbl absorption. In IGS or IFD, the rate of cyano-Cbl loading of the holo-transcobalamin will be lower. As with the Schilling test, it is conceivable that the addition of IF in a second test step could distinguish IGS from IFD, as the added IF should restore the Cbl absorption in IFD only. This test promises to be more specific than serum levels of total Cbl, methylmalonic acid, or homocysteine [10]. However, questions of sensitivity remain to be answered but such a test would streamline clinical diagnostics.

## Conclusions

Elucidating the genetic basis of inherited Cbl malabsorption has provided the tools to verify the diagnosis in over 80% of the cases on the molecular level. In addition, studying this rare phenotype has elucidated the mechanisms and pathway of Cbl uptake in great detail. Our study triples the number of Cbl malabsorption cases molecularly analyzed and provides a comprehensive overview of the genetic patterns that cause this genetically heterogenous disease. The mutational patterns we have identified should simplify genetic diagnostics.

## Competing interests

The authors have no competing financial interests.

## Authors' contributions

SMT conceived and designed the study, coordinated the research, and wrote the manuscript. ACS and ECB coordinated DNA sample collection and performed genetic counseling. SL helped to draft the manuscript. AdlC commented on the manuscript draft. All authors read and approved the final manuscript.

## Supplementary Material

Additional file 1 **Title: Description of all patients and families included in the study.** Description: Details and results of all patients and families included in the study. Click here for file

Additional file 2 **Title: Sequence alignments of*CUBN*,*AMN*, and*GIF*.** Description: Alignments of the three gene sequences using HomoloGene with missense mutations highlighted in red. Click here for file

## References

[B1] Watkins D Rosenblatt DS Inborn errors of cobalamin absorption and metabolism Am J Med Genet C Semin Med Genet 2011 157 33 44 21312325 10.1002/ajmg.c.30288

[B2] Watkins D Rosenblatt DS Update and new concepts in vitamin responsive disorders of folate transport and metabolism J Inherit Metab Dis 2011 35 665 670 22108709 10.1007/s10545-011-9418-1

[B3] Allen LH How common is vitamin B-12 deficiency? Am J Clin Nutr 2009 89 693S 696S 19116323 10.3945/ajcn.2008.26947A

[B4] Shinawi M Hyperhomocysteinemia and cobalamin disorders Mol Genet Metab 2007 90 113 121 17340683 10.1016/j.ymgme.2006.11.012

[B5] Whitehead VM Acquired and inherited disorders of cobalamin and folate in children Br J Haematol 2006 134 125 136 16846473 10.1111/j.1365-2141.2006.06133.x

[B6] Gräsbeck R Imerslund-Gräsbeck Syndrome (selective vitamin B12 malabsorption with proteinuria) Orphanet journal of rare diseases 2006 1 1 17 10.1186/1750-1172-1-17 PMC1513194 16722557

[B7] Rössler J Breitenstein S Havers W Late onset of Imerslund-Gräsbeck syndrome without proteinuria in four children of one family from the Lebanon Eur J Pediatr 2003 162 808 809 14593474 10.1007/s00431-003-1306-8

[B8] Overgaard UM Tanner SM Birgens HS Vitamin B12 deficiency in a 15-year old boy due to mutations in the intrinsic factor gene, GIF Br J Haematol 2010 150 369 371 20408840 10.1111/j.1365-2141.2010.08198.x PMC2914612

[B9] Gräsbeck R Tanner SM Juvenile selective vitamin B malabsorption: 50 years after its description-10 years of genetic testing Pediatr Res 2011 70 222 228 21623254 10.1203/PDR.0b013e3182242124 PMC3152595

[B10] Carmel R Green R Rosenblatt DS Watkins D Update on cobalamin, folate, and homocysteine Hematology (Am Soc Hematol Educ Program) 2003 62 81 14633777 10.1182/asheducation-2003.1.62

[B11] Schilling RF Intrinsic factor studies II. The effect of gastric juice on the urinary excretion of radioactivity after oral administration of radioactive vitamin B12 J Lab Clin Med 1953 42 860 866 13109347

[B12] Carmel R The disappearance of cobalamin absorption testing: a critical diagnostic loss J Nutr 2007 137 2481 2484 17951489 10.1093/jn/137.11.2481

[B13] Aminoff M Carter JE Chadwick RB Johnson C Gräsbeck R Abdelaal MA Broch H Jenner LB Verroust PJ Moestrup SK Mutations in CUBN, encoding the intrinsic factor-vitamin B12 receptor, cubilin, cause hereditary megaloblastic anaemia 1 Nat Genet 1999 21 309 313 10080186 10.1038/6831

[B14] Tanner SM Aminoff M Wright FA Liyanarachchi S Kuronen M Saarinen A Massika O Mandel H Broch H de la Chapelle A Amnionless, essential for mouse gastrulation, is mutated in recessive hereditary megaloblastic anemia Nat Genet 2003 33 426 429 12590260 10.1038/ng1098

[B15] Tanner SM Li Z Bisson R Acar C Oner C Oner R Cetin M Abdelaal MA Ismail EA Lissens W Genetically heterogeneous selective intestinal malabsorption of vitamin B12: founder effects, consanguinity, and high clinical awareness explain aggregations in Scandinavia and the Middle East Hum Mutat 2004 23 327 333 15024727 10.1002/humu.20014

[B16] Broch H Imerslund O Monn E Hovig T Seip M Imerslund-Gräsbeck anemia A long-term follow-up study. Acta Paediatr Scand 1984 73 248 253 10.1111/j.1651-2227.1984.tb09937.x 6741523

[B17] Gräsbeck R Gordin R Kantero I Kuhlbäck B Selective vitamin B12 malabsorption and proteinuria in young people Acta Med Scand 1960 167 289 296 13828999 10.1111/j.0954-6820.1960.tb03549.x

[B18] Imerslund O Idiopathic chronic megaloblastic anemia in children Acta Paediatr Scand 1960 1 115 13852753

[B19] Wahlstedt-Fröberg V Pettersson T Aminoff M Dugué B Gräsbeck R Proteinuria in cubilin-deficient patients with selective vitamin B(12) malabsorption Pediatr Nephrol 2003 18 417 421 12687456 10.1007/s00467-003-1128-y

[B20] Birn H The kidney in vitamin B12 and folate homeostasis: characterization of receptors for tubular uptake of vitamins and carrier proteins Am J Physiol Renal Physiol 2006 291 F22 F36 16760376 10.1152/ajprenal.00385.2005

[B21] Fyfe JC Madsen M Hojrup P Christensen EI Tanner SM de la Chapelle A He Q Moestrup SK The functional cobalamin (vitamin B12)-intrinsic factor receptor is a novel complex of cubilin and amnionless Blood 2004 103 1573 1579 14576052 10.1182/blood-2003-08-2852

[B22] Katz M Lee SK Cooper BA Vitamin B 12 malabsorption due to a biologically inert intrinsic factor N Engl J Med 1972 287 425 429 5044916 10.1056/NEJM197208312870902

[B23] Ament AE Li Z Sturm AC Perko JD Lawson S Masterson M Quadros EV Tanner SM Juvenile cobalamin deficiency in individuals of African ancestry is caused by a founder mutation in the intrinsic factor gene GIF Br J Haematol 2009 144 622 624 19036097 10.1111/j.1365-2141.2008.07496.x PMC2636683

[B24] Tanner SM Li Z Perko JD Oner C Cetin M Altay C Yurtsever Z David KL Faivre L Ismail EA Hereditary juvenile cobalamin deficiency caused by mutations in the intrinsic factor gene Proc Natl Acad Sci U S A 2005 102 4130 4133 15738392 10.1073/pnas.0500517102 PMC554821

[B25] Yassin F Rothenberg SP Rao S Gordon MM Alpers DH Quadros EV Identification of a 4-base deletion in the gene in inherited intrinsic factor deficiency Blood 2004 103 1515 1517 14576042 10.1182/blood-2003-07-2239

[B26] Al-Alami JR Tanner SM Tayeh MK de la Chapelle A El-Shanti H Homozygous AMN mutation in hereditary selective intestinal malabsorption of vitamin B12 in Jordan Saudi Med J 2005 26 1061 1064 16047053

[B27] Bouchlaka C Maktouf C Mahjoub B Ayadi A Sfar MT Sioud M Gueddich N Belhadjali Z Rebai A Abdelhak S Dellagi K Genetic heterogeneity of megaloblastic anaemia type 1 in Tunisian patients J Hum Genet 2007 52 262 270 17285242 10.1007/s10038-007-0110-0

[B28] Broides A Yerushalmi B Levy R Hadad N Kaplun N Tanner SM de la Chapelle A Levy J Imerslund-Grasbeck syndrome associated with recurrent aphthous stomatitis and defective neutrophil function J Pediatr Hematol Oncol 2006 28 715 719 17114957 10.1097/01.mph.0000243656.25938.7b

[B29] Densupsoontorn N Sanpakit K Vijarnsorn C Pattaragarn A Kangwanpornsiri C Jatutipsompol C Tirapongporn H Jirapinyo P Shah NP Sturm AC Tanner SM Imerslund-Grasbeck syndrome: New mutation in amnionless Pediatrics international : official journal of the Japan Pediatric Society 2012 54 e19 e21 22631584 10.1111/j.1442-200X.2011.03482.x

[B30] Garcia Jimenez MC Baldellou Vazquez A Calvo Martin MT Perez-Lungmus G Lopez Pison J Hereditary juvenile cobalamin deficiency due to mutations in GIF gene An Pediatr (Barc) 2008 69 56 58 18620679 10.1157/13124221

[B31] Hauck FH Tanner SM Henker J Laass MW Imerslund-Grasbeck syndrome in a 15-year-old German girl caused by compound heterozygous mutations in CUBN Eur J Pediatr 2008 167 671 675 17668238 10.1007/s00431-007-0571-3

[B32] Leunbach TL Johansen P Tanner SM Grasbeck R Helgestad J Homozygous mutation in the intrinsic factor gene in a child with severe vitamin B12 deficiency Ugeskrift for laeger 2011 173 2047 2048 21867658

[B33] Luder AS Tanner SM de la Chapelle A Walter JH Amnionless (AMN) mutations in Imerslund-Gräsbeck syndrome may be associated with disturbed vitamin B(12) transport into the CNS J Inherit Metab Dis 2008 10.1007/s10545-007-0760-2 18181028

[B34] Namour F Dobrovoljski G Chery C Audonnet S Feillet F Sperl W Gueant JL Luminal expression of cubilin is impaired in Imerslund-Grasbeck syndrome with compound AMN mutations in intron 3 and exon 7 Haematologica 2011 96 1715 1719 21750092 10.3324/haematol.2011.043984 PMC3208692

[B35] Siddiqui AH Ansari A Beech CM Shah NP Tanner SM Sarnaik SA Juvenile cobalamin deficiency in a 17-year-old child with autonomic dysfunction and skin changes J Pediatr Hematol Oncol 2012 34 140 142 22082743 10.1097/MPH.0b013e3182288249 PMC3302209

[B36] Storm T Emma F Verroust PJ Hertz JM Nielsen R Christensen EI A patient with cubilin deficiency N Engl J Med 2011 364 89 91 21208123 10.1056/NEJMc1009804

[B37] Liechti-Gallati S Schneider V Neeser D Kraemer R Two buffer PAGE system-based SSCP/HD analysis: a general protocol for rapid and sensitive mutation screening in cystic fibrosis and any other human genetic disease Eur J Hum Genet 1999 7 590 598 10439967 10.1038/sj.ejhg.5200338

[B38] den Dunnen JT Antonarakis SE Nomenclature for the description of human sequence variations Hum Genet 2001 109 121 124 11479744 10.1007/s004390100505

[B39] Tomsic J Guda K Liyanarachchi S Hampel H Natale L Markowitz SD Tanner SM de la Chapelle A Allele-specific expression of TGFBR1 in colon cancer patients Carcinogenesis 2010 31 1800 1804 20705955 10.1093/carcin/bgq165 PMC2950937

[B40] Adzhubei IA Schmidt S Peshkin L Ramensky VE Gerasimova A Bork P Kondrashov AS Sunyaev SR A method and server for predicting damaging missense mutations Nat Methods 2010 7 248 249 20354512 10.1038/nmeth0410-248 PMC2855889

[B41] Burset M Seledtsov IA Solovyev VV SpliceDB: database of canonical and non-canonical mammalian splice sites Nucleic Acids Res 2001 29 255 259 11125105 10.1093/nar/29.1.255 PMC29840

[B42] Rutsch F Gailus S Miousse IR Suormala T Sagne C Toliat MR Nurnberg G Wittkampf T Buers I Sharifi A Identification of a putative lysosomal cobalamin exporter altered in the cblF defect of vitamin B12 metabolism Nat Genet 2009 41 234 239 19136951 10.1038/ng.294

[B43] Kristiansen M Kozyraki R Jacobsen C Nexø E Verroust PJ Moestrup SK Molecular dissection of the intrinsic factor-vitamin B12 receptor, cubilin, discloses regions important for membrane association and ligand binding J Biol Chem 1999 274 20540 20544 10400683 10.1074/jbc.274.29.20540

[B44] Beech CM Liyanarachchi S Shah NP Sturm AC Sadiq MF de la Chapelle A Tanner SM Ancient founder mutation is responsible for Imerslund-Grasbeck Syndrome among diverse ethnicities Orphanet journal of rare diseases 2011 6 74 22078000 10.1186/1750-1172-6-74 PMC3226546

[B45] Kalantry S Manning S Haub O Tomihara-Newberger C Lee HG Fangman J Disteche CM Manova K Lacy E The amnionless gene, essential for mouse gastrulation, encodes a visceral-endoderm-specific protein with an extracellular cysteine-rich domain Nat Genet 2001 27 412 416 11279523 10.1038/86912

[B46] Sturm AC Baack EC Armstrong MB Schiff D Zia A Savasan S de la Chapelle A Tanner SM Hereditary Intrinsic Factor Deficiency in Chaldeans J Inherit Metab Dis 2012 in press 10.1007/8904_2012_133 PMC3575053 23430489

[B47] Ovunc B Otto EA Vega-Warner V Saisawat P Ashraf S Ramaswami G Fathy HM Schoeb D Chernin G Lyons RH Exome sequencing reveals cubilin mutation as a single-gene cause of proteinuria Journal of the American Society of Nephrology: JASN 2011 22 1815 1820 21903995 10.1681/ASN.2011040337 PMC3187182

[B48] Boger CA Chen MH Tin A Olden M Kottgen A de Boer IH Fuchsberger C O'Seaghdha CM Pattaro C Teumer A CUBN is a gene locus for albuminuria Journal of the American Society of Nephrology : JASN 2011 22 555 570 21355061 10.1681/ASN.2010060598 PMC3060449

[B49] Greibe E Fedosov S Nexo E The Cobalamin-Binding Protein in Zebrafish Is an Intermediate between the Three Cobalamin-Binding Proteins in Human PLoS One 2012 7 e35660 22532867 10.1371/journal.pone.0035660 PMC3331988

[B50] Johnston J Yang-Feng T Berliner N Genomic structure and mapping of the chromosomal gene for transcobalamin I (TCN1): comparison to human intrinsic factor Genomics 1992 12 459 464 1463480 10.1016/0888-7543(92)90435-u

[B51] Carmel R Mild transcobalamin I (haptocorrin) deficiency and low serum cobalamin concentrations Clin Chem 2003 49 1367 1374 12881454 10.1373/49.8.1367

[B52] Nielsen R Christensen EI Proteinuria and events beyond the slit Pediatr Nephrol 2010 25 813 822 20049615 10.1007/s00467-009-1381-9

[B53] He Q Madsen M Kilkenney A Gregory B Christensen EI Vorum H Hojrup P Schaffer AA Kirkness EF Tanner SM Amnionless function is required for cubilin brush-border expression and intrinsic factor-cobalamin (vitamin B12) absorption in vivo Blood 2005 106 1447 1453 15845892 10.1182/blood-2005-03-1197 PMC1895201

[B54] Reznichenko A Snieder H van den Born J de Borst MH Damman J van Dijk MC van Goor H Hepkema BG Hillebrands JL Leuvenink HG CUBN as a Novel Locus for End-Stage Renal Disease: Insights from Renal Transplantation PLoS One 2012 7 e36512 22574174 10.1371/journal.pone.0036512 PMC3344899

[B55] Lovblad K Ramelli G Remonda L Nirkko AC Ozdoba C Schroth G Retardation of myelination due to dietary vitamin B12 deficiency: cranial MRI findings Pediatr Radiol 1997 27 155 158 9028851 10.1007/s002470050090

[B56] Bjorke-Monsen AL Ueland PM Cobalamin status in children J Inherit Metab Dis 2011 34 111 119 20508991 10.1007/s10545-010-9119-1

[B57] Smith BT Mussell JC Fleming PA Barth JL Spyropoulos DD Cooley MA Drake CJ Argraves WS Targeted disruption of cubilin reveals essential developmental roles in the structure and function of endoderm and in somite formation BMC Dev Biol 2006 6 30 16787536 10.1186/1471-213X-6-30 PMC1533814

[B58] Strope S Rivi R Metzger T Manova K Lacy E Mouse amnionless, which is required for primitive streak assembly, mediates cell-surface localization and endocytic function of cubilin on visceral endoderm and kidney proximal tubules Development 2004 131 4787 4795 15342463 10.1242/dev.01341

[B59] Weyer K Storm T Shan J Vainio S Kozyraki R Verroust PJ Christensen EI Nielsen R Mouse model of proximal tubule endocytic dysfunction Nephrol Dial Transplant 2011 26 3446 3451 21926402 10.1093/ndt/gfr525

[B60] Carmel R Parker J Kelman Z Genomic mutations associated with mild and severe deficiencies of transcobalamin I (haptocorrin) that cause mildly and severely low serum cobalamin levels Br J Haematol 2009 147 386 391 19686235 10.1111/j.1365-2141.2009.07855.x

[B61] Hazra A Kraft P Lazarus R Chen C Chanock SJ Jacques P Selhub J Hunter DJ Genome-wide significant predictors of metabolites in the one-carbon metabolism pathway Hum Mol Genet 2009 18 4677 4687 19744961 10.1093/hmg/ddp428 PMC2773275

[B62] Hazra A Kraft P Selhub J Giovannucci EL Thomas G Hoover RN Chanock SJ Hunter DJ Common variants of FUT2 are associated with plasma vitamin B12 levels Nat Genet 2008 40 1160 1162 18776911 10.1038/ng.210 PMC2673801

[B63] Tanaka T Scheet P Giusti B Bandinelli S Piras MG Usala G Lai S Mulas A Corsi AM Vestrini A Genome-wide association study of vitamin B6, vitamin B12, folate, and homocysteine blood concentrations Am J Hum Genet 2009 84 477 482 19303062 10.1016/j.ajhg.2009.02.011 PMC2667971

[B64] Quadros EV Lai SC Nakayama Y Sequeira JM Hannibal L Wang S Jacobsen DW Fedosov S Wright E Gallagher RC Positive newborn screen for methylmalonic aciduria identifies the first mutation in TCblR/CD320, the gene for cellular uptake of transcobalamin-bound vitamin B(12) Hum Mutat 2010 31 924 929 20524213 10.1002/humu.21297 PMC2909035

[B65] Quadros EV Nakayama Y Sequeira JM The protein and the gene encoding the receptor for the cellular uptake of transcobalamin-bound cobalamin Blood 2009 113 186 192 18779389 10.1182/blood-2008-05-158949 PMC2614632

[B66] Beedholm-Ebsen R van de Wetering K Hardlei T Nexo E Borst P Moestrup SK Identification of multidrug resistance protein 1 (MRP1/ABCC1) as a molecular gate for cellular export of cobalamin Blood 2010 115 1632 1639 19897579 10.1182/blood-2009-07-232587

[B67] Shah NP Beech CM Sturm AC Tanner SM Investigation of the ABC transporter MRP1 in selected patients with presumed defects in vitamin B12 absorption Blood 2011 117 4397 4398 21511966 10.1182/blood-2010-12-322750

[B68] Frater-Schroder M Genetic patterns of transcobalamin II and the relationships with congenital defects Mol Cell Biochem 1983 56 5 31 6355816 10.1007/BF00228765

[B69] Haberle J Pauli S Berning C Koch HG Linnebank M TC II deficiency: avoidance of false-negative molecular genetics by RNA-based investigations J Hum Genet 2009 54 331 334 19373259 10.1038/jhg.2009.34

[B70] Rutsch F Gailus S Suormala T Fowler B LMBRD1: the gene for the cblF defect of vitamin B metabolism J Inherit Metab Dis 2011 34 121 126 20446115 10.1007/s10545-010-9083-9

[B71] Hardlei TF Morkbak AL Bor MV Bailey LB Hvas AM Nexø E Assessment of vitamin B(12) absorption based on the accumulation of orally administered cyanocobalamin on transcobalamin Clin Chem 2010 56 432 436 20040621 10.1373/clinchem.2009.131524 PMC10594690

