# Supplementary material for: Inherited cobalamin malabsorption. Mutations in three genes reveal functional and ethnic patterns
Source: Orphanet J Rare Dis. 2012 Aug 28;7:56. doi: 10.1186/1750-1172-7-56 (PMC3462684; doi:10.1186/1750-1172-7-56)
Supplement: Additional file 2 — Title: Sequence alignments ofCUBN,AMN, andGIF. Description: Alignments of the three gene sequences using HomoloGene with missense mutations highlighted in red. [file 1750-1172-7-56-S2.pdf]

Additional file 2

Sequence alignments of *CUBN*, *AMN*, and *GIF*.

Multi-species sequence alignments of the three gene sequences using HomoloGene with missense mutations highlighted in red.

HomoloGene:37434 (*CUBN*)

Protein Acc.	Organism
NP_001072.2	H.sapiens
XP_507675.2	P.troglodytes
NP_001003148.1	C.lupus
XP_601498.3	B.taurus
NP_001074553.1	M.musculus
NP_445784.1	R.norvegicus
XP_001235156.1	G.gallus
XP_002666724.1	D.rerio
NP_727348.2	D.melanogaster
XP_315526.4	A.gambiae
NP_506157.3	C.elegans

```
NP_001072.2           --------------------------------------------------
XP_507675.2           --------------------------------------------------
NP_001003148.1        --------------------------------------------------
XP_601498.3           --------------------------------------------------
NP_001074553.1        --------------------------------------------------
NP_445784.1           --------------------------------------------------
XP_001235156.1     1  MVEEEEEEDEDEEEGSDEEEKGLLSFDVFGQPPLNQRTNPSDDSIPGDLG  50
XP_002666724.1        --------------------------------------------------
NP_727348.2           --------------------------------------------------
XP_315526.4           --------------------------------------------------
NP_506157.3           --------------------------------------------------

NP_001072.2        1  ------------------------------------MMNMSLPF------  8
XP_507675.2        1  -------------------------------------MNMSLPF------  7
NP_001003148.1     1  ---------------------------------------MSSPF------  5
XP_601498.3        1  ---------------------------------------MSSLF------  5
NP_001074553.1     1  ---------------------------------------MASHF------  5
NP_445784.1        1  ---------------------------------------MSSQF------  5
XP_001235156.1    51  QQPIEQLKVRSSNVQCPDPTLCQAHIPQDHKLNQGMVAAAQATFSVDIFD  100
XP_002666724.1        --------------------------------------------------
NP_727348.2        1  -----------------------------------MEGAARSRL------  9
XP_315526.4           --------------------------------------------------
NP_506157.3        1  -----------------------------------------------MIP  3

NP_001072.2        9  -LWSLLTLLIFAEVNGEAGELELQRQKRSINLQQPRMATERGNLVFLTGS  57
XP_507675.2        8  -LWSLLTLLTFAEVNGEAGELELQRLKRSINLQQPRMATERGNLVFLTGS  56
NP_001003148.1     6  -LWSLIILLTFAESNGEAGGFELQRQKRSIDFQQPRMATERGNLVFLVGS  54
XP_601498.3        6  -LGSLVILLTFAASYSEDGGLALQRQKRNIDLQQPRMTAERGNLVFLTGS  54
NP_001074553.1     6  -LWGFVTLLMVPGLDGETGTPEQKLQKRIADLHQPRMTTEEGNLVFLTSS  54
NP_445784.1        6  -LWGFVTLLMIAELDGKTGKPEQRGQKRIADLHQPRMTTEEGNLVFLTSS  54
XP_001235156.1   101  DLFCIVALLLLAGLSCESNHYEKKRQKRNIYDEQPRLSSEQGNLVFHAGS  150
XP_002666724.1        --------------------------------------------------
NP_727348.2       10  -LLCWTLLAIITDTWPIAEGF----------VNSPKIISKDGNLIFESGA  48
XP_315526.4        1  ------------------MTP----------YNRAKILATNGHIIIESAQ  22
NP_506157.3        4  NLQLFLSLILFGLLNHVSSIDYEEIHIEDSYETRSRILMVDGNVWLHAGK  53
```

```
NP_001072.2      58   AQNIEFRTGSLGKIKLN-DEDLSECLHQIQKN------------------   88
XP_507675.2      57   AQNIEFRTGSLGKIKLN-DEDLSECLHQIQKN------------------   87
NP_001003148.1   55   AQNIEFRTGSLGKIKLN-EEDLGECLHQIQKN------------------   85
XP_601498.3      55   AQNIEFRTGSLGRIKLN-DEDVGECFHQIQKN------------------   85
NP_001074553.1   55   AQNIEFRTGSLGKIKLN-DDDLGECLHQIQRN------------------   85
NP_445784.1      55   TQNIEFRTGSLGKIKLN-DEDLGECLHQIQRN------------------   85
XP_001235156.1  151   SKNIEFRTGPLGKIKLN-EEDLAEMFSQMREN------------------   181
XP_002666724.1        --------------------------------------------------
NP_727348.2      49   NRNISFRLSGNSRLTINEELDVMELL--LATSGSKKRSGGKDDDFVDARE   96
XP_315526.4      23   DRNITFYMKGSGHLNIG-GLSIEQVLRTLSKMMIDGRPSTDSQPFLPGGS   71
NP_506157.3      54   DKNITFKTTGNGRIYVD-ETDVSKLPDIASFKQLVGRIENTSQMFTTLKS   102

NP_001072.2      89   -KED--------IIELKGSAIGL-----------PQNISSQIYQLNSK--   116
XP_507675.2      88   -KDD--------IIELKGSAIDL-----------PQNISSQIYQLNSK--   115
NP_001003148.1   86   -KED--------ITDLKRSAVNV-----------PQNISSQIHQLNSK--   113
XP_601498.3      86   -KDD--------ITNLKRTTVGL-----------PQNISNQIHQLDSK--   113
NP_001074553.1   86   -KDD--------IIDLKRNTTGL-----------PQNILSQVHQLNSK--   113
NP_445784.1      86   -KDD--------IIDLRKNTTGL-----------PQNILSQVHQLNSK--   113
XP_001235156.1  182   -KAE--------IQELKKAS-GV-----------SQNVSHQVSMLASK--   208
XP_002666724.1        --------------------------------------------------
NP_727348.2      97   LADQ--------LADFNRRAFGANGLSAMLRV--QQNRTRGSMALLRR--   134
XP_315526.4      72   VADQ--------VQLLATYVSGPYGLIKRVETLEQQSSSRNVTQMNPR--   111
NP_506157.3     103   RQDTQTSAMRVVLNSAKHYLVALRNMTLEVNILKKWKTDKMARDMRRRGL   152

                                                      Gly145Glu
NP_001072.2     117   LVDLERKFQGLQQTVDK-------KVCSSNPCQNGGTCLNLHDS-FFCIC   158
XP_507675.2     116   LVDLERKFQGLQQTVDK-------KVCSSNPCQNGGTCLNLHDS-FFCIC   157
NP_001003148.1  114   LVDLERKFQSLQQTVDK-------KVCSSNPCQNGGTCLNLHDS-FFCIC   155
XP_601498.3     114   LADLERKLQSLQQTLDR-------KVCSSNPCQHGGTCLNLPDS-FFCMC   155
NP_001074553.1  114   LVDLERDFQSLQQNVER-------KVCSSNPCHNGGTCVNLHDS-FICIC   155
NP_445784.1     114   LVDLERDFQNLQQNVER-------KVCSSNPCLNGGTCVNLHDS-FVCIC   155
XP_001235156.1  209   VTSLDMKLQSLEQTLQR-------KACSSNPCENSGTCVNSLDG-FFCLC   250
XP_002666724.1        --------------------------------------------------
NP_727348.2     135   ---FQTRLRALENRVDRMKTDLEANSCASGPCENGGTCYNTYTG-FRCQC   180
XP_315526.4     112   IGALARRVRNVETKVALLMSTLQVNRCTSGPCQNGGTCIAQYDS-FMCLC   160
NP_506157.3     153   LVKMTRQIIGINKLIAI-------NACDPNKCSNGGTCIPSFGAKFTCLC   195

NP_001072.2     159   PPQWKGPLCSADVNECEIYSGTPLSCQNGGTCVNTMGSYSCHCPPETYGP   208
XP_507675.2     158   PPQWKGSLCSADVNECEIYSGTPLSCQNGGTCVNTMGSYSCHCPPETYGP   207
NP_001003148.1  156   PSQWKGPLCSVDVNECQIYSGTPLGCQNGATCENTAGSYSCLCSPETHGP   205
XP_601498.3     156   PSQWKGPLCSVDVNECEIYSGTPLGCQNGATCINTAGSYSCLCSPETHGP   205
NP_001074553.1  156   PSQWKGLFCSEDVNECVLYAGTPFGCQSGSTCVNTMGSFRCDCTPDTYGP   205
NP_445784.1     156   PSQWKGLFCSEDVNECVVYSGTPFGCQSGSTCVNTVGSFRCDCTPDTYGP   205
XP_001235156.1  251   PSNWRGLRCSEDINECQIYAGTALGCQNGATCVNTPGSYSCSCTPETYGP   300
XP_002666724.1    1   -----GPTCAVDVNECQVLAGTPLGCQNGATCNNTPGSYTCTCTPEWYGP   45
NP_727348.2     181   RSAFEGTKCEMDVNECALYEGTDLGCQNGGQCQNHFGTYSCLCQPGWHGM   230
XP_315526.4     161   PSNWEGQTCTTDVNECALFAGTDLGCQNGATCKNIHGGYTCMCPDGWRGI   210
NP_506157.3     196   PPHFTGTTCEADIDECSVYNGTTAGCQNNGTCINNRGGFECQCQSGYHGS   245

                                   Cys225Ser
NP_001072.2     209   QCASKYDDCEGGSV-ARCV-HGICEDLMREQAGEPK-----YSCVCDAGW   251
XP_507675.2     208   QCASKYDDCEGGSV-ARCV-HGICEDLMREQAGEPK-----YSCICDAGW   250
NP_001003148.1  206   QCASKYDDCEGGSK-ALCV-HGICEDLVRVKADEPK-----YNCICDAGW   248
XP_601498.3     206   QCASKYDDCEGGSK-ARCM-HGICEDLVRVKAGEPE-----YSCICDAGW   248
NP_001074553.1  206   QCASKYNDCEQGSQ-QLCK-HGICEDLQRVYHGQLR-----FNCICDAGW   248
NP_445784.1     206   QCASKYNDCEQGSK-QLCK-HGICEDLQRVHHGQPN-----FHCICDAGW   248
```

```
XP_001235156.1    301   RCASKFDDCQGGSQ-TLCE-HGTCVDAERDTPNKPK-----YHCICDTGW   343
XP_002666724.1     46   HCTSRYDDCAGGSQ-DLCV-HGLCIDSDRVNPNEPK-----YKCICDAGW   88
NP_727348.2       231   HCTQRKADCSQSSAWELCG-HGSCVPS----ADDAG-----YRCICEPGW   270
XP_315526.4       211   HCNTRSQDCATAGA-DLCG-HGTCVQA----KD--G-----YRCICDQGW   247
NP_506157.3       246   LCQYHMSACSKTF--ELCGPHGHCIESIVDPTGQSSSDTTTYKCICDWGF   293

NP_001072.2       252   M--FSPNSPACTLDRDECSFQPGPCS--TLVQCFNTQGSFYCGACPTGWQ   297
XP_507675.2       251   M--SSPNSPACTLDRDECSFQPGPCS--TLVQCFNTQGSFYCGACPTGWQ   296
NP_001003148.1    249   T--SPLNSSACVLDIDECNLQHAPCS--PLVQCFNTQGSFYCGACPTGWQ   294
XP_601498.3       249   K--APSNSTACTLDVDECNLWPAPCS--ELVRCFNTPGSFYCGACPTGWQ   294
NP_001074553.1    249   T--TLPNGISCTEDKDECSLQPSPCS--EHAQCFNTQGSFYCGACPKGWQ   294
NP_445784.1       249   T--TPPNGISCTEDKDECSLQPSPCS--EHAQCFNTQGSFYCGACPKGWQ   294
XP_001235156.1    344   M--SPPGSPACSADIDECSLPNPPCSQNPRVQCHNTLGSYSCDPCPTGWQ   391
XP_002666724.1     89   T--SPPGVAACTADIDECSLPTKPCSTNPPVECFNTLGSFYCGACPSGWQ   136
NP_727348.2       271   K--TNGLTPICGEDVDECSDSAAH--KPCSTSCINLPGSFTCAPCPAGLT   316
XP_315526.4       248   K--TNGLTPACSVDVDECSESKPHCSKDPEVSCINLPGSFVCGPCPAGYS   295
NP_506157.3       294   KVSSDKNNPTC-VDVNEC--ESNPCH--PGVDCINLPGSFVCSGCPKGYK   338

                                                           Pro337Leu
NP_001072.2       298   GNGYICEDINECEINNGGCSVAPPVECVNTPGSSHCQACPPGYQGDGRVC   347
XP_507675.2       297   GNGYVCEDINECEINNGGCSVAPPVECVNTPGSSHCQACPPGYQGDGRVC   346
NP_001003148.1    295   GNGYSCQDIDECKINNGGCSVVPPVMCVNTLGSYHCQACPPGYQGDGRVC   344
XP_601498.3       295   GNGYICEDINECEINNGGCSVAPLVECINTHGSYHCHSCPPGYQGDGRVC   344
NP_001074553.1    295   GNGYQCQDINECEINNGGCSQAPLVPCLNTPGSFTCGNCPAGFSGDGRVC   344
NP_445784.1       295   GNGYECQDINECEINNGGCSQAPLVPCLNTPGSFSCGNCPAGFSGDGRVC   344
XP_001235156.1    392   GNGYSCQDIDECESDNGGCSTAPMVQCINTIGSFRCGVCPPGYEGDGQTC   441
XP_002666724.1    137   GNGYSCQDVDECATNNGGCSTSPFVPCLNTMGSFHCGQCPPGYEGDGKTC   186
NP_727348.2       317   GNGVSCRDLDECQTNNGGCSLSPKVDCINTYGSYHCGECPVGWTGDGRKC   366
XP_315526.4       296   GNGFYCVDIDECETNNGGCSTSPSVQCINTRGSYRCGNCPAGYTGDGRTC   345
NP_506157.3       339   TDGNVCIDVNECEGEIRVCS--PLSKCHNTLGSYYCDSCPTGYSGDGGNC   386

NP_001072.2       348   ------------TLTDICSVSNGGCHPDASCSS--TLGS-LP--LCTCLP   380
XP_507675.2       347   ------------TLIDICSVSNGGCHPDASCSS--TLGS-LP--LCTCLP   379
NP_001003148.1    345   ------------TVIDICSVNNGGCHPEASCSS--VLGS-LP--LCTCLP   377
XP_601498.3       345   ------------TLVDRCSVNNGGCHPQASCSL--ALGS-LP--LCTCLP   377
NP_001074553.1    345   ------------TPLDICSIHNGGCHPDATCSSSSVLGSLLP--VCTCPP   380
NP_445784.1       345   ------------TPVDICSIHNGGCHPEATCSSSPVLGSFLP--VCTCPP   380
XP_001235156.1    442   ------------TQVDSCSINNGGCHPSATCTSTPGL---MP--FCSCSP   474
XP_002666724.1    187   ------------TQADICSTNNGGCFPLATCTS--TPGSTIP--LCTCPP   220
NP_727348.2       367   ERSPQDIDIPAGQTPRTCPAGNNPCYPTASCF----LISGTT--SCRCPM   410
XP_315526.4       346   --------LAHGN---RCTQG--LCHPMARCVD---YGSAVP--NCICLP   377
NP_506157.3       387   ------------VKDDSCVKNK--CHKLATCKVTDDGYSAVGDYTCYCPD   422

NP_001072.2       381   GYTGNGYGPNGCVQLSNIC-LSHPCLN-GQCIDT-VSGYFCKCDSGWTGV   427
XP_507675.2       380   GYTGNGYGPDGCVQLSNIC-LSHPCLN-GQCIDT-VSGYFCKCDSGWTGV   426
NP_001003148.1    378   GYTGNGYGPNGCAQLSDTC-LSHPCLN-GQCIET-VSGYLCKCESGWAGI   424
XP_601498.3       378   GYTGNGYGPNGCVQLSNIC-LSRPCVN-GQCIET-VSGYVCKCESGWTSV   424
NP_001074553.1    381   GYTGNGYGSNGCVRLSNMC-SRHPCVN-GQCIET-VSSYFCKCDSGWFGQ   427
NP_445784.1       381   GYTGNGYGSNGCVRLSNIC-SRHPCVN-GQCIET-VSSYFCKCDSGWSGQ   427
XP_001235156.1    475   GYTGSGYGPNGCSPLTDICQLQNPCAN-GQCLAV-TSGYFCLCNAGWTGS   522
XP_002666724.1    221   GYVGNGYGPTGCTQISDICGTSNPCVN-GQC-------------------   250
NP_727348.2       411   GMVGTGYGPNGCVNGTTTNCKENPCLNGGICLFAGPSNYTCLCPIGFRPP   460
XP_315526.4       378   GYIGSGFGPNGCYRSSMNPCASAPCRNGGTCTKIDAQNYSCACPPGTNPP   427
NP_506157.3       423   GYVGDGIGEEGCVKSASNVCQNHNCVNAGKCKPTSDTEYKCECEAGFLGK   472

NP_001072.2       428   NCTENINECLSNPCLNGGTCV--DGVDSFSCECTRLWTGALCQVPQQVCG   475
XP_507675.2       427   NCTENINECLSNPCLNGGTCV--DGVDSFSCECTRLWTGALCQVPQQVCG   474
```

```
NP_001003148.1    425   NCTENINECLSNPCFNGGTCV--DGVNAFSCECTRFWTGFLCQIPQQVCG   472
XP_601498.3       425   NCTENINECLSNPCLNGGTCV--DGVNAFSCECTHFWTGFLCHIPQEVCG   472
NP_001074553.1    428   NCTENINECVSNPCLNGGTCI--DGVNGFTCDCTSSWTGYYCQTPQAACG   475
NP_445784.1       428   NCTENINDCSSNPCLNGGTCI--DGINGFTCDCTSSWTGYYCQTPQAACG   475
XP_001235156.1    523   NCTENIDECISNPCQNGGSCT--DGVNGYTCECTSAWTGPQCQTAQQACG   570
XP_002666724.1          --------------------------------------------------
NP_727348.2       461   ICEPQPSPCDQHPCKNGGRCRPTTSGDLFVCQCLPGYRGRLCETRFSSCN   510
XP_315526.4       428   NCMRTTSPCESNPCQNGGTCVGSSGTRNFLCRCPAGYTGLRCQTPTRTCG   477
NP_506157.3       473   FC-EKTSPCQTNPCKNGGTCI--AVENSAYCDCPEHFFGRACEEEEEHCG   519

NP_001072.2       476   ESLSGINGSFSY--RSPDVGYVHDVNCFWVIKTEMGKVLRITFTFFR--L   521
XP_507675.2       475   ESLSGINGSFSY--RSPDVGYVHDVNCFWVIKTEMGKVLRITFTFFR--L   520
NP_001003148.1    473   GSLSGMDGSFSY--MSPDVGYVHDVNCFWVIRTEDRKVLRITFTFFQ--L   518
XP_601498.3       473   ESLSGMNGSFSF--VSPDVAYAHDINCFWVIHTEEGKVLRITFTFFQ--L   518
NP_001074553.1    476   GILSGTQGTFAY--QSPNDTYVHNVNCFWVVRTDEEKVLHITFTFFD--L   521
NP_445784.1       476   GILSGTQGTFAY--HSPNDTYIHNVNCFWIVRTDEEKVLHVTFTFFD--L   521
XP_001235156.1    571   GYLSGSRGTFSYPNNPSSQHYDTGVSCAWVIQTASNKILHITFPFFH--L   618
XP_002666724.1    251   ------------------------------------EILRITFXFFD--I   262
NP_727348.2       511   GMLSAQSGRLRY--PPEGTGYEHNAQCAWVIRTNESLVVNVTFNSFD--V   556
XP_315526.4       478   GIRFQMSGTLRY--PEFNGTYNHNARCAWLIKTNETQVLNVTFTQFS--L   523
NP_506157.3       520   SHFTHSSGNYTFDLQRSNKTELSICDFVFNIPAANSAVV-MTFTEFDKFT   568

NP_001072.2       522   ES---MDNCPHE--FLQVYDGDSSSAFQLGRFCGSS-----LPHE--LLS   559
XP_507675.2       521   ES---MDNCPHE--FLQVYDGDSSSAFQLGRFCGSS-----LPHE--LLS   558
NP_001003148.1    519   ES---VNNCPHE--FLQIHDGDSSAALQLGRFCGSV-----LPHE--LLS   556
XP_601498.3       519   EP---TNNCPHE--FLQIHDGDSSAAHQLGRFCGSS-----PPQE--LLS   556
NP_001074553.1    522   ES---ASNCPRE--YLQIHDGDSSADFPLGRYCGST-----PPQG--VHS   559
NP_445784.1       522   ES---ASNCPRE--YLQIHDGDSSADFPLGRYCGSR-----PPQG--IHS   559
XP_001235156.1    619   EA---STDCNSD--FLQIHDGASASMHMLGKYCGSN-----PPAE--LFS   656
XP_002666724.1    263   EN---SASCNFD--FLQVHDGESASAFVIGKYCGSA-----APAE--LFS   300
NP_727348.2       557   ED---STECRFD--WLQINDGRSAAAQIIGRYCGNH-----LPHGGNIVS   596
XP_315526.4       524   ENPVSSGECKYD--WLQIHDGRTSAAQIIGRFCGNE-----LPRGGNFQS   566
NP_506157.3       569   QEGSGPTDCAKTDANLTLYDGPEDSSSEFATFCGDSHSVHAPLSDTPITM   618

NP_001072.2       560   SDNALYFHLYSEHLRNGRGFTVRWETQQPECGGILTG-PYGSIKSPGYPG   608
XP_507675.2       559   SDNALYFHLYSEHLRNGRGFTVRWETQQPECGGILTG-TYGSIKSPGYPG   607
NP_001003148.1    557   SNNALYFHLYSEHFRSGRGFTIRWETQQPECGGILMG-TYGSIKSPGYPG   605
XP_601498.3       557   SDNALYFHLYSEHLRSERGFTIRWETQLPECGGMLTG-TYGSIKSPGYPG   605
NP_001074553.1    560   SANSLYFHLYSEYIKRGRGFTARWEAKLPECGGILTG-NYGSITSPGYPG   608
NP_445784.1       560   SANALYFHLYSEYIRSGRGFTARWEAKLPECGGILTD-NYGSITSPGYPG   608
XP_001235156.1    657   SHNSLYFWFHSNHAVTAGGFTVQWDSRDPECGGELTA-TYGSISSPGYPG   705
XP_002666724.1    301   SHNSLYFWFRSDHSVSAGGFTVAWQSQAPVCGGQLTN-TYGDIKSPGYPG   349
NP_727348.2       597   SGNQLYLWFRSDNSTAKEGFDLTWNSMEPQCGGRLNFETHGTLASPGSPG   646
XP_315526.4       567   THNMLYLWFRSDNATAHDGFQLRWESIDPVCGGTIAAVSHGLIASPGTPG   616
NP_506157.3       619   TSTGAMLRFRG----TQGSFTIKWETVERKCGYRSSK-PEGIISVPQNHQ   663

                                                             Arg651Gly
NP_001072.2       609   NYPPGRDCVWIVVTSPDLLVTFTFGTLSLEHHD--DCNKDYLEIRDG--P   654
XP_507675.2       608   NYPPGRDCVWIVVTSPDLLVTFTFGTLSLEHHD--DCNKDYLEIRDG--P   653
NP_001003148.1    606   NYPPGRDCVWQVVTSPDLLITFTFGTLSLEHHD--DCSKDYLEIRDG--P   651
XP_601498.3       606   KYPPGRDCVWRVITSPDLLITFTFGTLSLEHHD--DCSKDYLEIRDG--P   651
NP_001074553.1    609   NYPPGRDCVWNLLVSPGSLITFTFGTLSLESHN--DCSKDYLEIRDG--P   654
NP_445784.1       609   NYPPGRDCVWQVLVNPNSLITFTFGTLSLESHN--DCSKDYLEIRDG--P   654
XP_001235156.1    706   NYPVNRDCFWTISTNPGLLITFAFGTLSLEHHE--NCSYDYLEIRDG--L   751
XP_002666724.1    350   NYPPNRDCYWTVNVNPGLLITFAFGTLSLEHHD--NCNFDFLEIRDG--L   395
NP_727348.2       647   NYPKNRDCRWQLVAPTTKRIKLTFFSLQLEQHA--NCNFDYVLIKDS--I   692
XP_315526.4       617   NYPPNRDCKWYLQAPQGRRLQFTFFTMKIEVHE--TCGFDYLEITDG--L   662
```

```
NP_506157.3         664   DIV----CEWFISAPGGKIIEVTIPPVSMHSKDIEKCDQNSLEIYDGYAT   709

                                                     Ile690Val
NP_001072.2         655   LYQDPLLGKFCTTFSVPPLQTTGPFARIHFHSDSQISD------QGFHIT   698
XP_507675.2         654   LYQDPLLGKFCTTFSVPPLQTTGPFARIHFHSDSQISD------QGFHIT   697
NP_001003148.1      652   LYQDPSLGKFCTTLSVPPLQTTGPFARVHFHSDNQIND------QGFHIT   695
XP_601498.3         652   LHQDPVLGKFCTTLSAPPLQTTGPFARIHFHSDNEIND------QGFLIT   695
NP_001074553.1      655   FHHDPILGKFCTSLSTPPLQTTGPAARIHFHSDSETSD------KGFHIT   698
NP_445784.1         655   FHQDPVLGKFCTSLSTPPLKTTGPAARIHFHSDSETSD------KGFHIT   698
XP_001235156.1      752   LSQDSVLGKYCSTGSPPPLQTTGPYAWIHFHSDDEVTD------KGFHIV   795
XP_002666724.1      396   LPEDSLLGKYCSTASPPPLQTTGPSAWIHFHSDFSISD------RGFHIT   439
NP_727348.2         693   --SGRELAKYCTTGAPAPLLLPTHLAEIHFHSDAEGSD------TGFQLH   734
XP_315526.4         663   RDEGTLLAKYCNTSHPPPLITPSNEATVHFHSDESGND------AGFQIH   706
NP_506157.3         710   YDKHRILETCSSTLESQVVRTTGPFLTVAFISNMLQSDAGLETIRGFVLK   759

NP_001072.2         699   YLTSPSDLRCGGNYTDP------EGELFL--PELSGPFTHTRQCVYMMKQ   740
XP_507675.2         698   YLTSPSDLRCGGNYTDP------EGELFL--PELSGPFTHTRQCVYMMKQ   739
NP_001003148.1      696   YLTSPSDLHCGGNYTDP------EG-LLS--SDLSGPFTHNRQCIYIIKQ   736
XP_601498.3         696   YLTSPSDLQCGGNYTDP------EG-LLF--ADLSGPFMHDRQCIYTIKQ   736
NP_001074553.1      699   YLTTPSDLYCGGNYTDT------EGELLL--PPLTGPFSHSRQCVYLISQ   740
NP_445784.1         699   YLTTQSDLDCGGNYTDT------DGELLL--PPLSGPFSHSRQCVYLITQ   740
XP_001235156.1      796   YTTSAADPTCGGNYTDS------EGVITS--PFWPNSFINSQQCIYIIRQ   837
XP_002666724.1      440   YTTSPSDPGCGGVFTET------EGIIIS--PNWPNNYAHNRQCIYIIRM   481
NP_727348.2         735   YSVEERVPGCGGVYTAK------EGTISE--SSTANTEPGGVSCEYEIHL   776
XP_315526.4         707   YAVVEGVPGCGGTYTQR------EGVISSPLSQTDNVYPNNLNCEYLIKQ   750
NP_506157.3         760   YKFTTPDRECGAEIDNDSNDFSFSGVIES--PNYGSLYPPNMDCTWKING   807

NP_001072.2         741   PQGE-----QIQINFTHVELQCQSD-------------------------   760
XP_507675.2         740   PQGE-----QIQINFTHVELQCQSD-------------------------   759
NP_001003148.1      737   PLGE-----QIQVNFTHVELEGQSS-------------------------   756
XP_601498.3         737   PPGE-----QIQVNFTVVELEGQSG-------------------------   756
NP_001074553.1      741   PQGE-----QIVINFTHVELESQRG-------------------------   760
NP_445784.1         741   AQGE-----QIVINFTHVELESQMG-------------------------   760
XP_001235156.1      838   PEDE-----KIHLNFTHLELESHAG-------------------------   857
XP_002666724.1      482   PRSE-----QVALNFTHMDLEIHTG-------------------------   501
NP_727348.2         777   AVGE-----QVVIQFARLELDP----------------------------   793
XP_315526.4         751   PVGS-----RVEIRFSKFHLEQSEA-------------------------   770
NP_506157.3         808   TLSNGSYSGDMVLKLTFDEYDVKSGFSANGPGMHYRSFRRLLPRNEVEYG   857

NP_001072.2         761   -------------------SSQNYIEVRDGE----TLL-GKVCGNGT---   783
XP_507675.2         760   -------------------SSQNYIEVRDGE----TLL-GKVCGNGT---   782
NP_001003148.1      757   -------------------CSQSHIEVRDDK----ILL-GKVCDNET---   779
XP_601498.3         757   -------------------CSHSYIEVRDDQ----TLL-GKVCGNET---   779
NP_001074553.1      761   -------------------CSHTFIEVGDHE----SLL-RKICGNET---   783
NP_445784.1         761   -------------------CSHTYIEVGDHD----SLL-RKICGNET---   783
XP_001235156.1      858   -------------------CSLNYIEVRDGDSEMSSLI-TRFCHSTV---   884
XP_002666724.1      502   -------------------CLFDFVEVRDGTGETDPLI-GKYCGNTL---   528
NP_727348.2         794   ---------------------LDCLEVLDITDEGGSILQEKICGSDASRL   822
XP_315526.4         771   -------------------CKFDYLEIFDGPSTEDPSL-GKFCG---DRM   797
NP_506157.3         858   IAPGFSRVFAYRNLFDMGTCTNDFLKIHDGD---GTLV-QETCNPRR---   900

                                                                Ser829Leu
NP_001072.2         784   -ISHIKSITN-SVWIRFKIDASVEKASFRAVYQVACGDELTGE-GVIRSP   830
XP_507675.2         783   -ISHIKSITN-SVWIRFKIDASVEKASFRAVYQVACGGELTGE-GVIRSP   829
NP_001003148.1      780   -LPHIKSIRN-HIWIRLKIDASLVRASFRAVYQVACGGELTGE-GVIRSP   826
XP_601498.3         780   -LSHIKSITN-SIWIRLKMDVSVVRASFSAVYQVACGGELTGE-GIIRSP   826
NP_001074553.1      784   -LFPIRSISN-NVWIRLRIDALVQKASFRADYQVACGGELRGE-GVIRSP   830
```

```
NP_445784.1          784    -LFPIRSVSN-KVWIRLRIDALVQKASFRADYQVACGGMLRGE-GFFRSP   830
XP_001235156.1       885    -ISPITSTSN-SLWIKFKSDASVQRASFRAVYQVACGGSLSGE-GTIHSP   931
XP_002666724.1       529    -PAPILSTTN-GLWIRFQSDSSVSRAGFRAMYELACGGTLSGT-GQIRTP   575
NP_727348.2          823    NPPTFTSEFN-RLKIKFY----ARAGSFQLNYRMACDYKLNNEQGTITSP   867
XP_315526.4          798    -PPLFTSTGN-ALLLKFHTDWSAPNPGFSLVYKIKCGGTFTDPAVELISP   845
NP_506157.3          901    -PPNVLTVNNPAAVLTFHSDSAEQGKGFRIQYEMLCEKRVNGN-GTIQTW   948

                                                                   Ser865Asn
NP_001072.2          831    FFPNVYPGERTCRWTIHQPQSQVILLNFTVFEI-------GSSAHCET--   871
XP_507675.2          830    FFPNVYPGERTCRWTIHQPQSQVILLNFTVFET-------GSSAHCET--   870
NP_001003148.1       827    FYPNVYPGERICRWTIHQPQSQVVILNFTAFGI-------ESSAHCDT--   867
XP_601498.3          827    FYPNVYPGERVCVWTIRQPQSQVVLLNFTAFDM-------GGSAHCDT--   867
NP_001074553.1       831    FYPNAYAGRRTCRWTISQPPREVVLLNFTDFQI-------GSSSSCDT--   871
NP_445784.1          831    FYPNAYPGRRTCRWTISQPQRQVVLLNFTDFQI-------GSSASCDT--   871
XP_001235156.1       932    YFPRMSSQPKTCEWIISQSVSKVVILNFIDFDI-------RNTTTCDS--   972
XP_002666724.1       576    FHPDPYPHNKVCEWVINQPEGYVVTLNFLTFDV-------EGSSTCAF--   616
NP_727348.2          868    GYPNLTRSDRICTYTISTATNTVISLKRIDFQLTNGESDDDDNDECLT--   915
XP_315526.4          846    SYPQMYPSDQLCDYVIHAPLGKAIVLDFQDFDF-----EKNSFPKCEL--   888
NP_506157.3          949    NFPNGGAAG-TCTYIIEAPKTHVISVRFLTIGL-----RVLPMSECFYTP   992

NP_001072.2          872    -------DYVEIGSSSIL-GSPENKKYCGTDIP----SFI-TSVYNFLYV   908
XP_507675.2          871    -------DYVEIGSSSIL-GSPENKKYCGTDIP----SFI-TSVYNFLYV   907
NP_001003148.1       868    -------DYIEIGSSSIL-GSPENKKYCGTDIP----LFI-TSVYNFLYV   904
XP_601498.3          868    -------DYVEVGSSSIL-GSLKNEKHCGADIP----SFI-TSVYNSLHV   904
NP_001074553.1       872    -------DYIEIGPSSVL-GSPGNEKFCGTNIP----SFI-TSVYNVLYV   908
NP_445784.1          872    -------DYIEIGPSSVL-GSPGNEKFCSSNIP----SFI-TSVYNILYV   908
XP_001235156.1       973    -------DYVEVRDGNNT-ESPLLGKYCGTAVP----SRV-QSTRNNLYI   1009
XP_002666724.1       617    -------DHVEVRDGPSS-SSPLIGRYCGVEMP----PML-ESTQKSMFI   653
NP_727348.2          916    -------TNLRINDGL---NRKILGPYCGKNQP----EENFVSETNYLQL   951
XP_315526.4          889    -------DYVELYDGLLPTNETLLGRYCSTKAP----PQT-ISSKNVLLL   926
NP_506157.3          993    NAVETYENYVEFSGGRTD-NALFNRRYVCARYPFVEGAWMSVSAARRLQI   1041

NP_001072.2          909    TFVKSSSTENHGFMAKFSAEDLA-----CGEILTESTGTIQSPGHPNV--   951
XP_507675.2          908    TFVKSSSTENHGFMAKFSAEDLA-----CGEILTESTGTIQSPGHPNV--   950
NP_001003148.1       905    IFVKSSSTENHGFMAKFSAADLA-----CGEILTESTGIIQSPGHPNI--   947
XP_601498.3          905    IFVKSSSADNHGFVAEFSSAALA-----CGKVLTESTGSIQSPGYPNI--   947
NP_001074553.1       909    TFVKSSSMENRGFMAMFSSEKLE-----CGKVLTESTGIIESPGHPNV--   951
NP_445784.1          909    TFVKSSSMENRGFTAKFSSDKLE-----CGEVLTASTGIIESPGHPNV--   951
XP_001235156.1      1010    KF-RASSLTNLGFRAQYWPLDTV-----CGESLTGSEGTITSPGFPDV--   1051
XP_002666724.1       654    QFKTDASVSNHGFTAEFGSAEQG-----CGETLTEPTGSFTSPGHPTE--   696
NP_727348.2          952    HLSTDVDSMGRGFK--FEYRALATGNDKCGGVHTRSGDHIRLPVHDDS--   997
XP_315526.4          927    RFVSDGSVSGRGFKGNFSFHDVS-----CGGVLMREDTIIRSPMIAETGK   971
NP_506157.3         1042    KVGSDGNPMFKGLSLEYKTSDVG-----CGGVFSSMTGTISSPNYPEK--   1084

NP_001072.2          952    YPHGINCTWHILVQPNHLIHLMFETFHLEF--HYNCTNDYLEVYDT----   995
XP_507675.2          951    YPHGINCTWHILVQPDHLIHLMFETFHLEF--HYNCTNDYLEVYDT----   994
NP_001003148.1       948    YPHGINCTWHILVQPGHLIHLIFRKFHLEF--HYNCTNDYLEVYDT----   991
XP_601498.3          948    YPHGISCTWHIIVQPGHLIHLRFREFHLEF--HYNCTKDYLEVYDT----   991
NP_001074553.1       952    YPSGVNCTWHIVVQRGQLIRLVFSSFYLEF--HYNCANDYLEVYDT----   995
NP_445784.1          952    YPRGVNCTWHVVVQRGQLIRLEFSSFYLEF--HYNCTNDYLEIYDT----   995
XP_001235156.1      1052    YPHGINCIWTINVQPGYLIRLTFTSFNLPF--HSSCRMDYLEIYDN----   1095
XP_002666724.1       697    YPHGANCTWYISVPPGHLIRLSFSYFNMEF--HTNCAYDYVDVYDN----   740
NP_727348.2          998    YAGEATCYWVIMAPANKAIRLHWNSFSLEN--AVDCIYDYLEIYDSLGAQ   1045
XP_315526.4          972    YQHDAQCEWIIVAPAGHAVQLTWNSFELEV--SAWCVYDYVQVFDN----   1015
NP_506157.3         1085    YQPHMHCVYNLYVSWSKTVKLTFDVFDLEVTPAKSCEYDRVEIYTS--YH   1132

NP_001072.2          996    DSE---TSLGRYCGKSIPPSLTSSGNSLMLVFVTDSDLAYEGFLINYEAI   1042
```

```
XP_507675.2         995   DSE---TSLGRYCGKSIPPSLTSSGNSLMLVFVTDSDLAYEGFLINYEAI   1041
NP_001003148.1      992   GSN---TYLGRYCGKSIPPSLTSSTNSLKLIFVADSDLAYEGFLINYEAT   1038
XP_601498.3         992   GSQ---TFLGRYCGKSIPPSLTSNDNSVRLTFVADSDLAYEGFLINYEAT   1038
NP_001074553.1      996   IAQ---TSLGRYCGKSIPPSLTSSSHSIKLIFVSDSALAHEGFSINYEAI   1042
NP_445784.1         996   AAQ---TFLGRYCGKSIPPSLTSNSNSIKLIFVSDSALAHEGFSINYEAI   1042
XP_001235156.1     1096   STM---QKLGRYCGRSIPPSLTSGGNVMMLYFVTDRSISSEGFSANYISL   1142
XP_002666724.1      741   GTALTGTLLGRFCGRSVPPSLTSTDSLMTVLLVSDSSLSAEGFSADYISI   790
NP_727348.2        1046   VNDERSKPLAKYCGNSVPEDLLSHSRQLVLKFVSDYSESDGGFDLTYTFE   1095
XP_315526.4        1016   -SSMANSLVGRYCGTEKPPAITSTGNMVTIRFVTDSSSSKDGFSLSFNFI   1064
NP_506157.3        1133   NETVHGELLGKFCGAMIPPSIYSTTNTMAVVFVSDRSVAGPGWNAKFEAV   1182

NP_001072.2        1043   SAATACLQDYTDDLGTFTSPNFPNNYPNNWECIYRITVRTGQLIAVHFTN   1092
XP_507675.2        1042   SAATACLQDYTDDLGTFTSPNFPSNYPNNWECIYRITVRTGQLIAVHFTN   1091
NP_001003148.1     1039   DASSACMEDYTENSGTFTSPNFPNNYPNNWKCIYRITVETSQQIALHFTN   1088
XP_601498.3        1039   DASAACLNDYTEESGTFTSPNFPGFYPNNLECIYRITVESSQQIALHFTN   1088
NP_001074553.1     1043   NASSVCLYDYTDNFGRLSSPNFPNNYPHNWNCVYRITVGLNQQIALHFTD   1092
NP_445784.1        1043   DASSVCLYDYTDNFGMLSSPNFPNNYPSNWECIYRITVGLNQQIALHFTD   1092
XP_001235156.1     1143   DASKVCSHNYNTETGVLTSPNYPNNYPVQTECIYTITVGINRQIVLRFTN   1192
XP_002666724.1      791   NATTDCSKVFRTSTGELSSPNYPDNYPTNRECVYRIIVEVNMQIMLNFTD   840
NP_727348.2        1096   DRAK-CGGHIHASSGELTSPEYPANYSAGLDCDWHLTGTIDHLLEIQVEN   1144
XP_315526.4        1065   DVEKSCGGNFFATSGIIRSPGWPKNYPSNKVCEWVITVPMGQQIMLLVHS   1114
NP_506157.3        1183   SRKTTCDFTLTAPSNNLIFDPQQLKFD---KCTYHIAVHENQRILIKMNN   1229

NP_001072.2        1093   FSLEEAI-GNYYTDFLEIRDGGYEKSPLLGIF------YGS--NLPPTII   1133
XP_507675.2        1092   FSLEEAI-GNYYTDFLEIRDGGYEKSPLLGIF------YGS--NLPPTII   1132
NP_001003148.1     1089   FALEEAIGGQCVADFVEIRDGGYETSPPLGTY------CGS--IPPPRII   1130
XP_601498.3        1089   FSLEDAIGSTCVADYVEIRNGGYENSPLLGQY------CGS--NLPPRII   1130
NP_001074553.1     1093   FALEDYFGPKCV-DFVEIRDGGFETSPLIGIY------CGS--VFPPRII   1133
NP_445784.1        1093   FTLEDYFGSQCV-DFVEIRDGGYETSPLVGIY------CGS--VLPPTII   1133
XP_001235156.1     1193   FTLEGNL--RCTEDYIEIRDGGYETSPYLGKY------CGS--GLPPVII   1232
XP_002666724.1      841   FQLEGF--SSCGFDYLEIRDGGYETSPLIGQY------CGT--NAPPIIV   880
NP_727348.2        1145   FELEQS--PNCSADYLEVRNGGGTDSPLIGRF------CGR--DIPARIP   1184
XP_315526.4        1115   FKMEKH--RICRFDGLTIRNGGTQNAPLIGNY------CGE--DNFNGTI   1154
NP_506157.3        1230   MSL------PCDKSSLMFRNGPSETSPPFSSLPPESEICTPKVNYMPVIR   1273

NP_001072.2        1134   SHSNKLWLKFKSDQIDTRSGFSAYWDGSSTGCGGNLTTSSGTFISPNYPM   1183
XP_507675.2        1133   SHSNKLWLKFKSDQIDTRSGFSAYWDGSSTGCGGNLTTSSGTFISPNYPM   1182
NP_001003148.1     1131   SHSNKLWLQFTSDFLGSGPGFSAYWDGSLTGCGGNITTPTGVFTSPSYPM   1180
XP_601498.3        1131   SHSNKLWLKFKSDFFGSGPGFSAYWDGSLTGCGGNLTTSTGTFTSPNYPM   1180
NP_001074553.1     1134   SHSNKLWLRFKSDTALTARGFSAYWDASSTGCGGNLTTPTGVLTSPNYPM   1183
NP_445784.1        1134   SHSNKLWLKFKSDAALTAKGFSAYWDGSSTGCGGNLTTPTGVLTSPNYPM   1183
XP_001235156.1     1233   SHGNKLWIKFVSDIFGTRKGFSAEWDGTSAGCGGTLTTSSGIFMSPNYPM   1282
XP_002666724.1      881   SHSNRLWMKFRSDHSLTYRGFQAHWDGTQTGCGGTLTTSSGGFTSPNYPL   930
NP_727348.2        1185   GFSHEMRLILHTDSAINGRGFRLRWRIFAFGCGGSLRSNMGAISSPRYPN   1234
XP_315526.4        1155   SFSHQLYLRFYSDSSRNYAGFMIEWDSATTGCGGILTSPRGSIISPNYPL   1204
NP_506157.3        1274   SFSNRVTIVYKS-INSEGSFFNLTYETITSGCGGRVDGLTGIVSAPQYPL   1322

                               Trp1193Gly
NP_001072.2        1184   PYYHSSECYWWLKSSHGSAFELEFKDFHLEHHPN----CTL---DYLAVY   1226
XP_507675.2        1183   PYYHSSECYWWLKSSHGSAFELEFKDFHLEHHPN----CTL---DYLAVY   1225
NP_001003148.1     1181   PYYHSSECYWLLKASHGSPFELEFEDFHLEHHPN----CTL---DYLAVY   1223
XP_601498.3        1181   PYYHSSECSWWLKASRGSPFLLEFEDFHLEYHPN----CSQ---DYLAVY   1223
NP_001074553.1     1184   PYYHSSECYWRLEASRGSPFLLEFQDFHLEHHPN----CSL---DYLAVF   1226
NP_445784.1        1184   PYYHSSECYWRLEASHGSPFELEFQDFHLEHHPS----CSL---DYLAVF   1226
XP_001235156.1     1283   PYYHSSECYWLLRGSRGTPFEIQFEQFHLEYHPN----CNF---DYLAVY   1325
XP_002666724.1      931   PYPANAECYWHIKTSAGSRIQLSFGDFHLEDTVD----CYF---DYLMVY   973
NP_727348.2        1235   SYPNMAHCEWRISLHPGSGISLLIEDLELEGLSN----CYY---DSVKIY   1277
```

```
XP_315526.4       1205   PYGQNALCTWRISMSQGSAIHIVFTDMDMESHKD----CQY---DYLVIY   1247
NP_506157.3       1323   GDKKNLKCDWTVAVALGNKVRFALTALDDLNSSDSGGFCPLFAANRIDFF   1372

                                                 Ser1250Phe
NP_001072.2       1227   DGPSSNSHLLTQLCGDEK----PPLIRSSGDSMFIKLRTDEGQQGR---G   1269
XP_507675.2       1226   DGPSSNSHLLTQLCGDEK----PPLIRSSGDSMFIKLRTDEGQQGR---G   1268
NP_001003148.1    1224   DGPSTSSHLLSQLCGNEK----PPVIRSTGDSMFLKFRTDEDQQGG---G   1266
XP_601498.3       1224   DGSSTSSHLLTRLCGNEK----PSVIRSSGDSMSLKLRTDEGQQGG---G   1266
NP_001074553.1    1227   DGPSTNSRLINKLCGDTP----PAPIRSSKDIVLLKLRTDAGQQGR---G   1269
NP_445784.1       1227   DGPTTNSRLIDKLCGDTT----PAPIRSNKDVVLLKLRTDAGQQGR---G   1269
XP_001235156.1    1326   DGNSSNAKQLGKFCGNQI----PQFINSSGDSVYIKLRTDSSVHGG---G   1368
XP_002666724.1     974   DGNNSNAHQLAKLCGNQI----PAPISSSRENMYVKLRTDSIIHTG---G   1016
NP_727348.2       1278   TGIKLPNQSPCKVLCKDDDLHNPLI-QLENNKGTIVFDSDASNTFR---G   1323
XP_315526.4       1248   DGIDTSGRKLGRFCSAETD---PIVLDTDTNHALIRMRTDETNQRR---G   1291
NP_506157.3       1373   DSALQGNQHLKRYCAKEMA---SEPITSDDNELIIKYVQSGGFQSKKIFG   1419

                                                 Pro1297Leu
NP_001072.2       1270   FKAEYRQTCENVVIVNQTYGILESIGYPNPY--SENQHCNWTIRATTGNT   1317
XP_507675.2       1269   FKAEYRQTCENVVIVNQTYGILESIGYPNPY--SENQHCNWTIRATTGNT   1316
NP_001003148.1    1267   FLAKYQQTCRNVVIVNRNYGILESIHYPNPY--SDNQRCNWTIQATTGNT   1314
XP_601498.3       1267   FLVKYQQTCDNVVIVNQTYGTLESIHYPNPY--SVNQRCNWTIQATTGNT   1314
NP_001074553.1    1270   FEINYRQTCDNVVIVNKTSGILESINYPNPY--DKDQRCNWTIQATTGNT   1317
NP_445784.1       1270   FEINFRQRCDNVVIVNKTSGILESINYPNPY--DKNQRCNWTIQATTGNT   1317
XP_001235156.1    1369   FLAKYKQVCHEVLTVNRSYGVLESLNYPNNY--PLGEHCRWTIQTTKGNT   1416
XP_002666724.1    1017   FLANYHSTCNGMLIANRSRGMIESLNYPNDY--PSHADCSWTIQAYMGNT   1064
NP_727348.2       1324   FRISYKANCIRNLTA--TTGTIESLNYMEPFWETIPINCSWTIRAPKGNR   1371
XP_315526.4       1292   FQLKYNILCRRNLTG--YGGVIESPNFPNEY--SASMDCRWTIRVPPGNK   1337
NP_506157.3       1420   FSGHFTTLCNGIVHEA-ISGSIQSPGYPYKV--YSNQFCTWTIKVPKGNR   1466

NP_001072.2       1318   VNYTFLAFDL----------EHHINCSTDYLELYDGP----RQ-------   1346
XP_507675.2       1317   VNYTFLAFDL----------EHHINCSTDYLELYDGP----RR-------   1345
NP_001003148.1    1315   VNYTFLAFEL----------ENHINCSTDYLELYDGP----RR-------   1343
XP_601498.3       1315   VNYTFLAFEL----------EYYTNCSTDYLELYDGP----QR-------   1343
NP_001074553.1    1318   VNYTFLEFDV----------ENYVNCSTDYLELYDGP----QR-------   1346
NP_445784.1       1318   VNYTFLGFDV----------ESYMNCSTDYVELYDGP----QW-------   1346
XP_001235156.1    1417   LSYSFTAFDV----------EDGSNCDRDYLKLYDGPNVQSNL-------   1449
XP_002666724.1    1065   INYTFIAFEV----------EQSDGCGYDHVKLYDGPNDQANL-------   1097
NP_727348.2       1372   VLVEVSHL------------ARHEQ----HVPTATMPGGLYIV-------   1398
XP_315526.4       1338   INLEFSHFDFESITQQTGSNATHQRCPFDYVELQEMGTGDLPV-------   1380
NP_506157.3       1467   IVATVHHFSISQKSYF----EGGINCITDMLKVDDTDLAEAEVTFKKTDY   1512

                                                       Thr1372Arg           Gly1390Ser
NP_001072.2       1347   -----MGRYCGVDLPPPGSTTSSKLQVLLLTDGVGRREKGFQMQWFVYGC   1391
XP_507675.2       1346   -----MGRYCGVDLPPPGSTTSSKLQVLLLTDGVGRREKGFQMQWFVYGC   1390
NP_001003148.1    1344   -----MGRYCGADMPPTGSTTGSKLQVLFYTDGVGHQEKGFQMQWFIHGC   1388
XP_601498.3       1344   -----MGRFCGTDIPPSGSTTGSKFHVLFYTDGVSHHEKGFQMQWFIQGC   1388
NP_001074553.1    1347   -----IGRYCGENIPPPGATTGSKLIVVFHTDGVDSGEKGFKMHWFIHGC   1391
NP_445784.1       1347   -----MGRYCGNNMPPPGATTGSQLHVLFHTDGINSGEKGFKMQWFTHGC   1391
XP_001235156.1    1450   -----IGTFCGQSLPLAGNSTGTSLHVEFYSDGLQARS-GFQMLWHTNGC   1493
XP_002666724.1    1098   -----IGTFCGNVPPPAGTTSGTSLHIVFRSD-MSISYRGFQMDWYQNGC   1141
NP_727348.2       1399   -----DGRNVQEIVTPQAMNISGEVLTVVHNA-SN---VNFQLDYRIDGC   1439
XP_315526.4       1381   -----ARRYCAN-KPPPIVSMGRSIDLIFHTD-SSGEQMGFRAEWSINGC   1423
NP_506157.3       1513   NITNSVNKYCDKAIPRVIRSRHNSMKLTYSSQGDPTNQ--FWLSWNTLGC   1560
```

HomoloGene:12804 (*AMN*)

```
Protein Acc.        Organism
NP_112205.2         H.sapiens
XP_510179.2         P.troglodytes
NP_001070509.1      B.taurus
NP_291081.2         M.musculus
XP_234547.4         R.norvegicus
XP_421379.2         G.gallus
XP_002661318.1      D.rerio

NP_112205.2        1   ----------------------------------MGVLGRVLLWLQLCAL   16
XP_510179.2        1   ----------------------------------MGVLGRVLLWLQLCAL   16
NP_001070509.1     1   ----------------------------------MGAPGLVLLWLQLCAL   16
NP_291081.2        1   ----------------------------------MGALGRVLLWLQLCAM   16
XP_234547.4        1   MCNIWAPPAPQPRPVTWGPRSEVRAGESLGGGGEMGALGRVLLWLQLCAL   50
XP_421379.2            --------------------------------------------------
XP_002661318.1     1   -----------MNSSNRQRQTKGRSAFLLPIPFMMALGHNVLLFFCISPF   39

                                             Thr41Ile           Leu59Pro
NP_112205.2       17   TQAVSKLWVPNTDFDVAANWSQNRTPCAGGAVEFPADKMVSVLVQEGHAV   66
XP_510179.2       17   TQAVSKLWVPNTDFDVAANWSQNRTPCAGGAVEFPADKMVSVLVQEGHAV   66
NP_001070509.1    17   TPAAYKVWVPNTNFDDATNWSQNRPPCAGAAVEFPADKTLSVLVREGHSI   66
NP_291081.2       17   TRAAYKLWVPNTSFDTASNWNQNRTPCAGDAVQFPADKMVSVLVRDSHAI   66
XP_234547.4       51   TRAAYKLWVPNTSFDTASNWNQNRTPCAGDAVQFPADKMVSVLVRDSHAI   100
XP_421379.2        1   -MAAYKKWIPNTNFETMSNWDKGRVPCASDVIHFEKDEVVSVFVRSTHAL   49
XP_002661318.1    40   ANALYKQWIPDTNFENATNWDKGSVPCGNDQVEFLANRKVSVYVETAHTI   89

NP_112205.2       67   SDMLLPLDGELVLASGAGFGVSDVGSHLDCGAGEPAVFRDSDRFSWHDPH   116
XP_510179.2       67   SDMLLPLDGELVLASGAGFGVSDVGSHLDCGAGEPAVFRDSDRFSWHDPH   116
NP_001070509.1    67   SDMLLPLDGEFVLASGAGFGAADAGSRPDCGPGARARFLDPDRFLWLDPR   116
NP_291081.2       67   SDMLLPLDGELVLASGAALSAAGGDSDPACNPGAPLLFRNPDRFSWLDPH   116
XP_234547.4      101   SDMLLPLNGEFVLASGAAFSAAGGDSDPACNPGAPLLFRNPDRFSWLDPH   150
XP_421379.2       50   TDMYLPLNGEFLLASGSGFTAFDGSWDPGCGSGAVLSFGNAEQHSWFNPE   99
XP_002661318.1    90   TGMSLPVDGELILASGAGFTVREGG-DPGCGSGVTAQFKDSESLKWFDPS   138

NP_112205.2      117   LWRSG------DEAPGLFFVDAERVPCRHDDVFFPPSASFRVGLGPGASP   160
XP_510179.2      117   LWRSG------DEAPGLFFVDAERVPCRHDDVFFPPSASFRVGLGPGASP   160
NP_001070509.1   117   LWRPG------DAGRGLFSVDAERVPCRHDDVVFPADASFRVGLGPGAGT   160
NP_291081.2      117   LWSSG------TQAPGLFSVDAERVPCSYDDVLFPRDGSFRVALGPGPNP   160
XP_234547.4      151   LWSSG------TQESGIFFVDAERVPCTYDDVLFPRNGSFRVALGPGPNP   194
XP_421379.2      100   LWQDVSSRVDLEPAGRIFLLDEERVPCQYDHVIFQPETSFRVNLDSSRRV   149
XP_002661318.1   139   LWEAATTIDDLHRGTFQFSVHEESVPCQNDDVVFRDATSFRVEISSDHD-   187

NP_112205.2      161   VRVRSISALGRTFTRDEDLAVFLASRAGRLRFHGPGALSVGPEDCADPSG   210
XP_510179.2      161   VRVRSISALGRTFTRDEDLAVFLASRAGRLRFHGPGALSVGPEDCADPSG   210
NP_001070509.1   161   VRVRSVQALGQTFARDEDLTAFLASRAGRLRFHGPGALSVDLEACADPSG   210
NP_291081.2      161   VHVRSVSAVGQTFSRDEDLTAFLASREGRLRFHGSGALRVGSQACTDASG   210
XP_234547.4      195   VHVRSVSAVGQTFTRDEDLAAFLASREGRLRFHGSGALRVGSQACTDATG   244
XP_421379.2      150   IPVQSISLMGQELSSAEAWAEYLQGPSAAWQFHGNATLQVTGTRCPHSSG   199
XP_002661318.1   188   VPVKSVSVLGKKFTSSSEFSQYLSSSSGKLQFHGTATISISSSGCGEISG   237

                                           Cys234Phe           Gly254Glu
NP_112205.2      211   CVCGNAEAQPWICAALLQPLGGRCPQAACHSALRPQGQCCDLCGAVVLLT   260
XP_510179.2      211   CVCGNAEAQPWICAALLQPLGGRCPQAACHSALRPQGQCCDLCGAVVLLT   260
NP_001070509.1   211   CVCGNAEAQPWICAALLQPLGGGCPQAACLEPLRPEGQCCDLCGAVVSLT   260
NP_291081.2      211   CVCGNAEMLPWICASLLQPLGGRCPQAACQDPLLPQGQCCDLCGAIVSLT   260
```

```
XP_234547.4        245   CVCGNAETLPWICASLLQPLGGRCPEATCQDPVRPQGQCCDLCGAIVSLT   294
XP_421379.2        200   CACGNSQDGDRICASLLRAEG--CPALSCHSPLRPLGHCCGVCGAVVTLD   247
XP_002661318.1     238   CICDNSRNREKICANV------RCDTLECKKPLHALGHCCNVCGALVYVQ   281

NP_112205.2        261   HGPAFDLERYRARILDTFLGLPQYHGLQVAVSKVPRSSRLRE-----ADT   305
XP_510179.2        261   HGPAFDLERYRARILDTFLGLPQYQGLQVAVSKVPRSSRLRE-----AAT   305
NP_001070509.1     261   HGPAFDLQQYRARLLHAFLALPQYQGLQVAVSKVPRPPGLREASDAKADT   310
NP_291081.2        261   HDPTFDLERYRARLLDLFLKQPQYQGLQVAVSKV-----LRD-----AHT   300
XP_234547.4        295   HDPTFDLERYRARLLDLFLKQPQYQGLQVAVSKV-----LRD-----AHT   334
XP_421379.2        248   FKPEFDLQQYRDRVVQAWLSLPRYAGVQMAISKVHRAQTFLGLLPRSSTP   297
XP_002661318.1     282   FSSSFNFESYRQRLQHLFLNTDKYRSVQMAMSKVSREQRLLGVIPFGATQ   331

NP_112205.2        306   EIQVVLV--ENGPETG-GAGRLARALLADVAENGEALGVLEATMRESGAH   352
XP_510179.2        306   EIQVVLV--ENGPETG-GAGRLARALLADVAENGEALGVLEATMRESGAH   352
NP_001070509.1     311   EIQVVLV--EAGPETG-GAGRLARALLADIAEHGEALGILSATARESGAP   357
NP_291081.2        301   EIQVVLV--ETEHATG-AAGQLGHALLQDAVAQGSVLGIVSATLRQSGKP   347
XP_234547.4        335   EIQVVLL--ETRPATG-AAGQLGHALLQDAVAQGSVLGIVSATLRQSGKP   381
XP_421379.2        298   VIQIVLVDGEAGTQTGTAAEQLAADIMGDIAQHGEALGIPGGSVEVATGS   347
XP_002661318.1     332   EIQVLLLDQKTGLESGNLAETLARDIMKDVHNHGSNVGISSAEFQASSGA   381

NP_112205.2        353   VWGSSA--------AGLAGGVAAAVLLALLVLLVAPPLLRRAGRL---R-   390
XP_510179.2        353   VWGSSA--------AGLAGGVAAAVLLALLVLLVAPPLLRRAGRL---R-   390
NP_001070509.1     358   VWGGSAAGLNAPARAGLAGGLAAAGLLVLTALLAGALLLRREGRL---R-   403
NP_291081.2        348   MTADSELNQSSSG-AGLAGGVAALVLLALLGTVL--LLLHRSGRL---R-   390
XP_234547.4        382   TAANSALDQNGSG-AGLAGGVAAVVLLALVGTVL--LLLHRSGRL---R-   424
XP_421379.2        348   ALGGQAAIHAS---RQIASGTVLGLLFALL-LLGALLYLHRKGKL---RL   390
XP_002661318.1     382   SSSDVAGNS-----AGVVAGAVLGCL-VILGLLAGFILLYRRGVVKLPRM   425

NP_112205.2        391   --------WRRHEAAAP--AGAPLGFRNPVFDVTASEELPLPRRLSLVPK   430
XP_510179.2        391   --------WRRHEAAAP--AGTPLGFHNPVFDVTASEELPLPRPLSLVPK   430
NP_001070509.1     404   --------WRRRDEAAPARAGAPLGFHNPVFYAADSAE-ALP-----APK   439
NP_291081.2        391   --------WRRHEDAEPVSAGLPLGFRNPIFDAIVFKQQPSVELPDSAQK   432
XP_234547.4        425   --------WRRHGDAEPTSAGLPLGFRNPIFDVMVFKQQASVEFADSTQK   466
XP_421379.2        391   QALHLPRLWNRAEDLNSPEPESGKGFDNPMFDV----ELPGAGSVEETLQ   436
XP_002661318.1     426   PSIPSLSKWRNGSDIGELGGPMDHGFDNPMFDKPTMMPEQPELYGSETMN   475

NP_112205.2        431   AAADSTSHSY--FVNPLF-AGAEAEA   453
XP_510179.2        431   AAADSTSHSY--FVNPLF-TGAEAEA   453
NP_001070509.1     440   SDQRSSSRSY--FLNPLF-GEAEAEA   462
NP_291081.2        433   VDILDIDTKFGCFVNPLFAGEAEAEA   458
XP_234547.4        467   VDILDIDTNFGCFVNPLFAGEAEAEA   492
XP_421379.2        437   EMASEGHQVF--YINPLY-DPSETET   459
XP_002661318.1     476   SVVLTKSGVH--FVNPVY-DETDFNG   498
```

HomoloGene:3773 (*GIF*)

**Protein Acc.**	**Organism**
NP_005133.2	H.sapiens
XP_508453.2	P.troglodytes
NP_001005759.1	C.lupus
XP_873322.1	B.taurus
NP_032144.2	M.musculus
NP_058858.1	R.norvegicus
XP_001233907.1	G.gallus
XP_001233885.1	G.gallus

```
                                                                     Ser46Leu
NP_005133.2        1   ----MAWFALYLLSLLWATAGTSTQTQSSCSVPSAQEPLVNGIQVLMENS   46
XP_508453.2        1   ----MAWFALYLLSLLWATAGTSTQTRSSCSVPSAQEPLVNGIQVLMENS   46
NP_001005759.1     1   ----MAWFSLHLLHLLWAAAGTSTWARSSCSVPQAAQHLVDGLQVLLEDS   46
XP_873322.1        1   ----MARAALQLLTLLWAATRTSTQTRSSCSVPSAEQPWVDGIQVIMENS   46
NP_032144.2        1   ----MAWLTLYLLSVLWAVAGTSTRAQSSCSVPPDQQPWVDGLQALMENS   46
NP_058858.1        1   MWKGMAWLSFYLLNVLWAVAGTSTRAQRSCSVPPDQQPWVNGLQLLMENS   50
XP_001233907.1         --------------------------------------------------
XP_001233885.1         --------------------------------------------------

NP_005133.2       47   VTSSAYPNPSILIAMNLAGAYNLKAQKLLTYQLMSSDNNDLTIGQLGLTI   96
XP_508453.2       47   VTSSAYPNPSILIAMNLAGAYNLKAQKLLTYQLMSSDTNDLTIGQLGLTI   96
NP_001005759.1    47   VSSAAPPNPSVLIAMNLAGALSAEARELLADRLGASDSAGLSVGQLALTI   96
XP_873322.1       47   VINSTSPNPSVLIAMNLAGAYNVEAQKLLTFDLMASDTADLTAGQLALTI   96
NP_032144.2       47   VTDSDFPNPSILIAMNLAGAYNVEAQKLLTYQLMASDSADLTSGQLALTV   96
NP_058858.1       51   VTESDLPNPSILIAMNLASTYNLEAQKLLTYQLMASDSADLTNGQLALTI   100
XP_001233907.1         --------------------------------------------------
XP_001233885.1         --------------------------------------------------

                    Met97Thr
NP_005133.2       97   MALTSSCRDPGDKVSILQRQMENWAPSSPNAEASAFYGPSLAILALCQKN   146
XP_508453.2       97   MALTSSCRDPGDKVSILQRQMENWAPSSPNAEASAFYGPSLAILALCQKN   146
NP_001005759.1    97   MALNSSCRDPGNKVSVLYGQMEAWPPSSPSAPAWTFYGPSLAVLALCQEH   146
XP_873322.1       97   MALTSSCRDPGDKVSTLRTQMENWTPSSLGSYASTFYGPSLAILALCQNN   146
NP_032144.2       97   MALTSSCRDPGSKVSTLLKKMENWSPSSPGAESSAFYGPGLAILALCQKS   146
NP_058858.1      101   MALTSSCRDPGSKVSILQKNMESWTPSNLGAESSSFYGPALAILALCQKN   150
XP_001233907.1         --------------------------------------------------
XP_001233885.1         --------------------------------------------------

                             Phe157Leu
NP_005133.2      147   SEATLPIAVRFAKTLLANSSPFNVDTGAMATLALTCMYNKIPVGSEEGYR   196
XP_508453.2      147   SEATLPIAVRFAKTLLANSSPFNVDTGAMATLALTCMYNKIPVGSEEGYR   196
NP_001005759.1   147   PGRALPVAARLAKILAAGLSPFNTDTGAMVTLALTCMYNKIPEGSEEGYR   196
XP_873322.1      147   PETTLPVAARFAKTLLASSSPFDVDAGAVATLALTCMYNRIPVGSEEGYR   196
NP_032144.2      147   SEATLPIAVRFAKTLMMEPSPFNVDTGAVATLALTCMYNKIPVGSQENYR   196
NP_058858.1      151   SEATLPIAVRFAKTLMMESSPFSVDTGAVATLALTCMYNRIPVGSQENYR   200
XP_001233907.1         --------------------------------------------------
XP_001233885.1         --------------------------------------------------

                                     Ile220Thr Ser225Arg Ala229Thr
NP_005133.2      197   SLFGQVLKDIVEKISMKIKDNGIIGDIYSTGLAMQALSVTPEPSKKEWNC   246
XP_508453.2      197   SLFGQVLKDIVEKISMKIKDNGIIGDIYSTGLAMQALSVTPEPPKKEWNC   246
NP_001005759.1   197   TLFSQVLKDVVENISMRIKDNGIIGDVYSTGLAMQALSVTPEPPNKEWDC   246
XP_873322.1      197   ALFAQLLKKIVEDISTRIRDNGLIGDVYSTGLAMQALSVTPERPNKQWDC   246
NP_032144.2      197   DLFGQALKAIVEKISLRIKADGIIGDIYSTGLAMQALSVTPEQPTKKWDC   246
```

```
NP_058858.1         201   DLFGQALKVIVDNISLRIKADGIIGDIYSTGLAMQALSVTPEQPTKEWDC   250
XP_001233907.1            --------------------------------------------------
XP_001233885.1            --------------------------------------------------

NP_005133.2         247   KKTTDMILNEIKQGKFHNPMSIAQILPSLKGKTYLDVPQVTCSPDHEVQP   296
XP_508453.2         247   KKTMDMILNEIKQGKFHNPMSIAQILPSLKGKTYLDVPQVTCSPDHEVQP   296
NP_001005759.1      247   KKTMDTILKEIEQGKFHNPMSIAQILPSLKGKTYLDVPYVSCSPGHQVQP   296
XP_873322.1         247   QQTMDTVLDEIKEGKFQNSMSIAQILPSLKGKTYLDVPQVSCSPDQEVQP   296
NP_032144.2         247   EKTMHTILNEIKQGKFQNPMSIAQILPSLKGKTYLDVPQVTCGPDHEVPP   296
NP_058858.1         251   EKTMYTILKEIKQGKFQNPMSIAQILPSLKGKTYLDVPQVTCGPDHEVPP   300
XP_001233907.1            --------------------------------------------------
XP_001233885.1            --------------------------------------------------
                                      Thr313Ile
NP_005133.2         297   TLPSNPGPGPTSASNITVIYTINNQLRGVELLFNETINVSVKSGSVLLVV   346
XP_508453.2         297   TLPSNPGPGPTSASNITVIYTINNQLRGVELLFNETINVSVKSGSVLLVV   346
NP_001005759.1      297   TLPSQPSPVPTSASNITVAYTINNQLKGVELVFNETIDVSVKDGSVLLVV   346
XP_873322.1         297   TQPNFPSPVPTSASNITVVYTISNQLRGVELLFNVTISVSVKKGSVLLVV   346
NP_032144.2         297   TLTDYPTPVPTSVSNITVIYTINNQLRGVDLLFNVTIEVSVKSGSVLLAV   346
NP_058858.1         301   TLTDYPTPVPTSISNITVIYTINNQLRGVDLLFNVTIEVSVKSGSVLLAV   350
XP_001233907.1        1   ----------MAGSNITVNYTITNEVRGKH--FSYSTEVEVPAGSVLLVV   38
XP_001233885.1        1   ------------GSNITVNYTITNEVRGKH--FSYSTEVEVPAGSVLLVV   36

NP_005133.2         347   LEEAQRKN-PMFKFETTMTSWGLVVSSINNIAENVNHKTYWQFLSGVTPL   395
XP_508453.2         347   LEEAQRKN-PMFKFETTMTSWGLVVSSINNIAENVNHKTYWQFLSGVTPL   395
NP_001005759.1      347   LEEAQRRN-PMFKFVTTMTSWGLVVSSINNIAESVHDRTYWQFLSGKTPL   395
XP_873322.1         347   LEEAQRRN-PKFKFETRMTSWGLEVSSINNIAGNVNDKTYWQFLSGKTPL   395
NP_032144.2         347   LEEAQRKN-SMFKFETTMTSWGLIVSSINNIAENVNHKTYWEFLSGKTPL   395
NP_058858.1         351   LEEAQRRN-HMFKFETTMTSWGLIVSSINNIAENVKHKTYWEFLSGKTPL   399
XP_001233907.1       39   LEEAQKSNKTIFSFKTESKPWGPVVVSIHGLDESVADKTFWQFFSGDDVL   88
XP_001233885.1       37   LEEAQKSNKTIFSFKTESTFWGPMVVSIHGLAASENDRTFWQFFSGDDVL   86

                                  Glu408Lys
NP_005133.2         396   NEGVADYIPFNHEHITANFTQY   417
XP_508453.2         396   NEGVADYIPFNHEHITANFTQY   417
NP_001005759.1      396   NEGVADYTPRDHEHITANFTQY   417
XP_873322.1         396   IEGVADYIPFNHEHITANFTQY   417
NP_032144.2         396   DEGVAYYIPFNHEHITANFTQY   417
NP_058858.1         400   GEGVAYYIPFNYEHITANFTQY   421
XP_001233907.1       89   PEGVGTYKPQDGEHIRAVFSIY   110
XP_001233885.1       87   PEGVGTYKPQDGEHIRAVFSIY   108
```
